# Mitochondrial and nuclear disease panel (Mito‐aND‐Panel): Combined sequencing of mitochondrial and nuclear DNA by a cost‐effective and sensitive NGS‐based method

**DOI:** 10.1002/mgg3.500

**Published:** 2018-11-08

**Authors:** Angela Abicht, Florentine Scharf, Stephanie Kleinle, Ulrike Schön, Elke Holinski‐Feder, Rita Horvath, Anna Benet‐Pagès, Isabel Diebold

**Affiliations:** ^1^ Medical Genetic Center Munich Munich Germany; ^2^ Department of Neurology, Friedrich‐Baur‐Institute Klinikum der Ludwig‐Maximilians‐Universität München Munich Germany; ^3^ Newcastle upon Tyne Hospitals NHS Trust Newcastle upon Tyne UK

**Keywords:** heteroplasmy detection, high‐throughput sequencing, mitochondrial and nuclear disease panel, mitochondrial disorders, mtDNA‐server, NUMTs

## Abstract

**Background:**

The diagnosis of mitochondrial disorders is challenging because of the clinical variability and genetic heterogeneity of these conditions. Next‐Generation Sequencing (NGS) technology offers a robust high‐throughput platform for nuclear and mitochondrial DNA (mtDNA) analyses.

**Method:**

We developed a custom Agilent SureSelect **Mito**chondrial **a**nd **N**uclear **D**isease Panel (Mito‐aND‐Panel) capture kit that allows parallel enrichment for subsequent NGS‐based sequence analysis of nuclear mitochondrial disease‐related genes and the complete mtDNA genome. Sequencing of enriched mtDNA simultaneously with nuclear genes was compared with the separated sequencing of the mitochondrial genome and whole exome sequencing (WES).

**Results:**

The Mito‐aND‐Panel permits accurate detection of low‐level mtDNA heteroplasmy due to a very high sequencing depth compared to standard diagnostic procedures using Sanger sequencing/SNaPshot and WES which is crucial to identify maternally inherited mitochondrial disorders.

**Conclusion:**

We established a NGS‐based method with combined sequencing of the complete mtDNA and nuclear genes which enables a more sensitive heteroplasmy detection of mtDNA mutations compared to traditional methods. Because the method promotes the analysis of mtDNA variants in large cohorts, it is cost‐effective and simple to setup, we anticipate this is a highly relevant method for sequence‐based genetic diagnosis in clinical diagnostic applications.

## 1 INTRODUCTION

Mitochondrial disease is highly heterogeneous in cause and features; therefore, traditional single‐gene testing strategies have had limited diagnostic success (McCormick, Place, & Falk, 2013). Due to clinical variability and large number of both nuclear and mitochondrial genes in which mutations can occur, parallel analysis of mtDNA and nuclear DNA within a routine diagnostic test is of great advantage in diagnosing mitochondrial diseases at the molecular level. Given the heteroplasmy of mitochondrial genomes, as well as the sequences of mitochondrial origin in the nuclear genome (NUMTs, nuclear mitochondrial DNAs), the dual analysis of genomic and mtDNA variants in a single NGS approach is challenging. The fact that subject DNA samples typically contain >100× copies of mtDNA molecules compared to nuclear DNA molecules is known to introduce bias in the dual sequence enrichment with the current targeted‐capture protocols (Calvo et al., 2012). In addition, deeper sequencing coverage of mtDNA compared to nuclear DNA is necessary to reliably detect low‐level heteroplasmic variants (Falk et al., 2012). While a mean coverage depth of 120× is usually enough for detection of nuclear DNA variants, a minimal depth of >1,000× reads may be acceptable for the detection of low‐heteroplasmic variants (Jennings et al., 2017; Matthijs et al., 2016), meaning that much more sequencing reads have to be generated for mtDNA compared to nuclear DNA. Although recent studies have demonstrated the efficacy of NGS technologies for the genetic diagnosis of inherited mitochondrial diseases (Calvo et al., 2012; Falk et al., 2012, 2015 ; Guo, Li, Li, Shyr, & Samuels, 2013; Legati et al., 2016; Picardi & Pesole, 2012; Vasta, Merritt, Saneto, & Hahn, 2012), these methods do not overcome the limitations and cause ineffectiveness in terms of variant detection accuracy, turnaround time, and costs for separate mtDNA and nuclear DNA analysis. Here, we developed a NGS‐based method with combined sequencing of the complete mtDNA and nuclear genes causing mitochondrial diseases and compared this method with the traditional Sanger sequencing and whole exome sequencing (WES).

## 2 MATERIALS AND METHODS

### 2.1 Patients and controls

DNA samples were extracted from peripheral blood (2–4 ml EDTA) of controls and of 17 patients with confirmed pathogenic mtDNA variants and controls. Informed consent was obtained from all participants and approved by local institutions (2018–027).

### 2.2 Selection of nuclear genes

The Nuclear Disease Panel (ND‐Panel) targets the coding sequences of 1,564 nuclear genes with a known disease‐associated phenotype in humans. These 1,564 nuclear genes are the combined aggregate of smaller phenotype‐related gene panels (gene sets of 2 to >400 genes) designed to address the most common neurological, neuromuscular, syndromic, and neurodevelopmental phenotypes in a clinical diagnostic setting. Phenotypes that may suggest underlying mitochondrial etiology include epilepsy, encephalopathy, Leigh syndrome, myopathy, hepatopathy, external ophthalmoplegia, optic atrophy, ataxia, dystonia, and stroke‐like episodes. In summary, 308 genes of the entire panel are contained in MitoCarta v2.0, an inventory of 1,158 human and mouse genes encoding proteins with strong support of mitochondrial localization (Calvo, Clauser, & Mootha, 2016) (Supporting information Table S1). The included MitoCarta v.2.0 genes are known to be associated with a mitochondrial disease phenotype in humans—thus confirming or strongly supporting a mitochondrial disorder in case of a pathogenic variant.

### 2.3 Sample preparation

For panel enrichment, approximately 1.5 µg genomic DNA of patients was required. Targeted enrichment was performed with two SureSelectXT Custom Kits (Agilent Biosciences): The mtDNA Panel (Mito‐Panel) which targets the complete sequence of the mitochondrial DNA and the Nuclear Disease Panel (ND‐Panel) which targets the coding sequences of 1,564 nuclear genes reported in human diseases, including 308 genes extracted from MitoCarta v2.0. Biotinylated RNA oligonucleotides (baits), targeting the entire mtDNA region and the coding exons of 1,564 nuclear genes, were designed (SureSelect 2× tiling across the target hg19/GRCh37 and the rCRS/NC_012920.1 mitochondrial genome). Both bait designs were ordered separately (Agilent Biosciences).

Given the 1–2 log natural excess of mtDNA genomes to the nuclear genome, blending of mtDNA capture baits will be necessary to ensure optimal coverage output of both genomes. First, performance of Mito‐Panel was tested in three negative control DNA samples (patients with no known pathogenic variant in the mtDNA sequence) at a ratio of 1:10, 1:20, 1:50, and 1:100 bait dilution. Sample preparation and hybridization was done in triplicates. Second, performance of nuclear DNA and mtDNA parallel sequence capture was tested at various mito:ND bait molar ratios.

**Figure 1:**
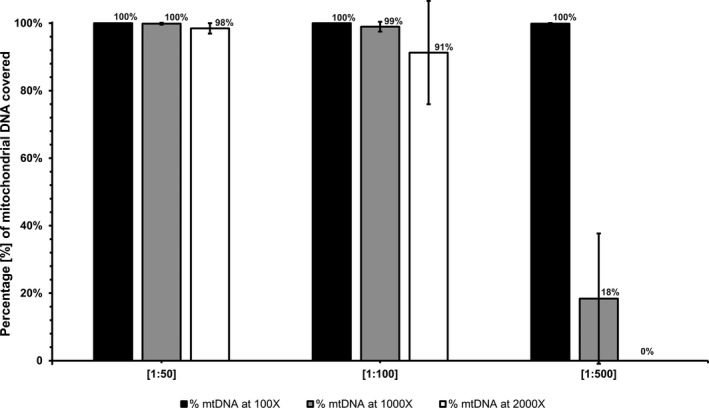
Evaluation of the capture efficiency of mtDNA when Nuclear Disease Panel (ND‐Panel) and Mito‐Panel are blended (=Mito‐aND‐Panel) at different concentrations. The validation cohort comprises 72 control samples including both mutation negative and positive distinct patients and two control DNAs of the Coriell repositories (NA12889, RM8398). The total of 72 samples was tested subdivided into three different sample preparation batches of three different molar ratios of mito:ND baits (batch A—dilution [1:50], batch B—dilution [1:100], and batch C—dilution [1:500]). Each sample batch was sequenced in independent sequencing runs. The height of the columns indicates the percentage of mtDNA covered by a minimum number of reads. Black‐colored column: minimum coverage of 100 reads, 100× coverage; gray‐colored column: minimum coverage of 1,000 reads, 1,000× coverage; white‐colored column: minimum coverage of 2,000 reads, 2,000× coverage. All data are expressed as arithmetic mean ± standard deviation of the samples

The validation cohort comprises 72 control samples including both mutation negative and positive distinct patients and two control DNAs of the Coriell repositories (NA12889, RM8398). The total of 72 samples was tested subdivided into three different sample preparation batches of three different molar ratios of mito:ND baits, namely batch A—dilution 1:50, batch B—dilution 1:100, and batch C—dilution 1:500 (Figure 1). The validation cohort comprised 68 negative control samples from patients and two control DNAs of the Coriell repositories (NA12889, RM8398) both with no pathogenic variant in the mtDNA sequence, which were prepared in duplicate, in order to confirm the repeatability of the method. Each sample batch was sequenced in independent sequencing runs (Figure 1).

Additional 17 positive control samples (human blood DNAs of patients with a pathogenic variant in the mtDNA sequence) were analyzed with the 1:50 mito:ND bait dilution (Table 1A,B), and the results were compared to the results from conventional Sanger sequencing. Additionally, 11 of these positive control DNA samples were compared to the results of whole exome sequencing (WES). WES and mtDNA enrichment was performed with the SureSelect Human All Exon v6 Kit (Agilent) following the manufacturer's instructions. All sample capture libraries were prepared as described in the SureSelectXT Target Enrichment System for Illumina Paired‐End Multiplexed Sequencing Library enrichment protocol.

**Table 1:** **(**A + B) Comparison of the mtDNA variants detected by different diagnostic procedures (Sanger Sequencing/SNaPshot analysis, Mitochondrial and Nuclear Disease Panel; Mito‐aND‐Panel and Whole Exome Sequencing; WES)

mtDNA Nucleotide Change	Gene	OMIM	Predicted Protein Change		% Heteroplasmy Sanger/SNaPshot		% Heteroplasmy Mito‐aND‐Panel		% Heteroplasmy WES
(A)									
m.3243A > G	*MT‐TL1*	* 590,050	Not applicable		10		10		
m.3243A > G	*MT‐TL1*	* 590,050	Not applicable		10		12.6		
m.3460G > A	*MT‐ND1*	* 516,000	Not applicable		10		12.6		
m.3243A > G	*MT‐TL1*	* 590,050	Not applicable		5		3.8		
m.3243A > G	*MT‐TL1*	* 590,050	Not applicable		5		4.6		
m.3243A > G	*MT‐TL1*	* 590,050	Not applicable		Not detected		4.5		
(B)									
m.8993T > G	*MT‐ATP6*	**+** 516,060		p.Leu156Arg	100	99		100	
m.8993T > G	*MT‐ATP6*	**+** 516,060		p.Leu156Arg	70	75		75	
m.8344A > G	*MT‐TK*	* 590,060		Not applicable	60	66		75	
m.3243A > G	*MT‐TL1*	* 590,050		Not applicable	60	65		64	
m.8993T > G	*MT‐ATP6*	**+** 516,060		p.Leu156Arg	70	60		100	
m.3243A > G	*MT‐TL1*	* 590,050		Not applicable	55	42		40	
m.8993T > G	*MT‐ATP6*	**+** 516,060		p.Leu156Arg	40	25		20	
m.3243A > G	*MT‐TL1*	* 590,050		Not applicable	20	17		not detected	
m.3243A > G	*MT‐TL1*	* 590,050		Not applicable	10	10.5		not detected	
m.3243A > G	*MT‐TL1*	* 590,050		Not applicable	5	4.5		not detected	
m.3243A > G	*MT‐TL1*	* 590,050		Not applicable	not detected	3.5		not detected	

Note

(A + B) A total of 17 positive control samples from patients with known mitochondrial DNA pathogenic variants (NC_012920.1): *MT‐ATP6* (m.8,993T > G), *MT‐TK* (m.8344A > G), or *MT‐TL1* (m.3243A>G) were enriched with 1:50 bait dilution and subsequently sequenced by next‐generation sequencing (NGS). The level of variant heteroplasmy in this cohort had been previously determined by Sanger sequencing and SNaPshot analysis. (B) 11 of these positive control samples were additionally tested by WES. OMIM accession number for the genes is included.

### 2.4 Sanger sequencing and SNaPshot

Sanger sequencing and single‐nucleotide allele‐specific primer extension (SNaPshot) analysis were used to determine the level of heteroplasmy in controls with pathogenic mtDNA variants (Cassandrini et al., 2006).

### 2.5 High‐throughput sequencing

Sequencing sample pools of mtDNA‐only (12 patient libraries per sequencing run) was performed on an Illumina MiSeq system Nano Flowcell (Illumina, San Diego, CA) as 150 bp paired‐end runs using v2.0 SBS chemistry. Sequencing of the Mito‐aND‐Panel and WES was carried out on an Illumina NextSeq 500 system (Illumina, San Diego, CA) as 150 bp paired‐end runs using v2.0 SBS chemistry.

### 2.6 Bioinformatics pipeline

#### 2.6.1 Detection of SNVs

The human reference genome hg19 was adapted by exchanging the outdated human mitochondrial reference sequence NC_001807.4 with the rCRS/NC_012920.1 sequence. Sequencing reads were aligned to this modified reference sequence using BWA (v0.7. 13‐r1126) with standard parameters. Duplicate reads and reads that did not map in proper pairs were removed. Statistics on coverage and sequencing depth on the clinical targeted regions (i.e., RefSeq coding exons and ±5 intronic region and complete mtDNA sequence rCRS/NC_012920.1) were calculated with a custom script. SNV and INDEL calling on the nuclear genes was conducted using SAMtools (v1.3.1) with subsequent coverage and quality‐dependent filter steps. Variant annotation was performed with snpEff (v4.2) and Alamut‐Batch (v1.4.4). Only variants (SNVs/small INDELs) in the coding and flanking intronic regions (±5 bp) were evaluated. The BAM files containing the mitochondrial sequencing data were analyzed separately with the mtDNA‐Server (v1.0.6) which detects both homoplasmic and heteroplasmic variants down to 1%, as well as possible contaminations based on haplogroup inconsistencies (Weissensteiner et al., 2016). Further, it tags positions in low complexity regions (LCR) and every variant is annotated with the number of known polymorphic nuclear mitochondrial insertions (NUMTs) and their location in the nuclear genome (Dayama, Emery, Kidd, & Mills, 2014).

#### 2.6.2 Interpretation of mitochondrial DNA Copies (NUMTs) in sequenced nuclear genomes

Variants located at known polymorphic nuclear mitochondrial insertions (NUMTs) were tagged by the mtDNA‐Server (Weissensteiner et al., 2016) in all patients. All these variants were carefully evaluated regarding their molecular effect and presence in the MITOMAP databases to assess possible pathogenicity or probable NUMTs of so far unknown NUMT regions.

#### 2.6.3 Sensitivity and specificity

Sensitivity and specificity of detecting nuclear variants in HapMap individuals (NA12889, NA12890, RM8398) was estimated using independent genotype training data obtained from the 1,000 Genomes and National Institute of Standards and Technology (NIST). Genotype concordance was assessed at 5,623 targeted sites.

Sensitivity and specificity of SNV detection in mtDNA via the mtDNA‐Server is being estimated based on the analysis of the validation cohort by comparing results from Sanger sequencing, SNaPshot, and NGS. Sanger sequencing is estimated to detect around 10% to 20% mutated DNA in a background of normal DNA (Tsiatis et al., 2010). The limit to detect heteroplasmic mtDNA variants may be as low as 6% (Irwin et al., 2009). The level of heteroplasmy detected by SNaPshot analysis, which is used for targeted verification or exclusion of individual variants (Cassandrini et al., 2006), is as low as 5% (Naue, Sanger, Schmidt, Klein, & Lutz‐Bonengel, 2011).

#### 2.6.4 Cost efficacy

The method combines testing for mutations of the mtDNA, and testing for mutations of frequent disease‐causing genes in the nuclear genome. Costs of the custom‐made targeted‐enrichment panel (SureSelect Tier 5 Panel, Agilent, Santa Clara, CA, USA) addressing the 1,564 nuclear‐encoded genes are lower compared to an entire whole exome or whole genome enrichment. Although the target enrichment does not cover all theoretically possible disease‐causing mutations of the nuclear genome, it enables the analysis of a large number of disease‐causing genes with the quality required for diagnostic analysis (Matthijs et al., 2016) using a NextSeq (Illumina, San Diego, CA) sequencing platform. The additional mtDNA baits (SureSelect Tier 1 Panel, Agilent, Santa Clara, CA, USA) increase the costs of enrichment to only about 1%. Furthermore, the combination of both methods in one technical approach leads to considerable savings in personnel costs and turnaround time.

## 3 RESULTS

### 3.1 Experimental evaluation of dual enrichment of mitochondrial and nuclear DNA

First, we experimentally evaluated the capture efficiency of the Mito‐Panel with the SureSelectXT method. Enrichment of three human blood DNA samples, each by one of four different dilutions of mito baits (1:10, 1:20, 1:50, and 1:100), resulted in high sequencing depth across the entire mtDNA genome regardless the oligo‐bait dilution factor. All data are expressed as standard deviation (*SD*). Average raw reads per sample were 1.79 × 10^4^ (*SD*: 7.7%). Briefly, mean sequencing depth for mtDNA of three different DNA samples in triplicates was 1,435 (*SD*: 12%) for 1:10, 1,421 (*SD*: 7.2%) for 1:20, 1,277 (*SD*: 0.8%) for 1:50, and 1,299 (*SD*: 8.1%) for 1:100 dilution. Mean capture repeatability between dilutions was >95% (*SD*: 2.8%).

Second, we evaluated the capture efficiency of nuclear and mtDNA genes when ND‐Panel and Mito‐Panel are blended (=Mito‐aND‐Panel) at different concentrations (Figure 1). Given the 1–2 log natural excess of mtDNA genomes to the nuclear genome, we sought to assess the optimal output of nuclear versus mtDNA genome by manually blending capture baits at different molar ratios. The aim of this experiment was to retain the ability to detect low‐level mtDNA variant heteroplasmy by high sequencing depth, but also maintain the diagnostic accuracy of variant detection in the nuclear genes by achieving sufficient sequencing depth of the ND‐Panel. Subsequently, we tested a total of 72 samples for this capture method subdivided into three different sample preparation batches of three different molar ratios of mito:ND baits (batch A—1:50, batch B—1:100, and batch C—1:500) as described above (Figure 1). Average raw reads per sample were 44 × 10^6^ (*SD* 17.9%). A mean sequencing depth of 7,373 (*SD* 18.1%)—batch A, 4,882 (*SD* 33.4%)—batch B, and 769 (*SD* 21.7%)—batch C was achieved on the complete mtDNA genome. The mean sequencing depth of the targeted regions of the ND‐Panel genes was 591 (*SD* 17.2%)—batch A, 666 (*SD* 12.5%)—batch B, and 491 (*SD* 7.8%)—batch C. All dilutions (batch A—1:50, batch B—1:100, batch C—1:500) showed a mean coverage of >99% of at least 30× for the coding regions of the ND‐Panel genes.

Specifically, 99.6% (*SD* 0.1%) of the clinical target of the 308 captured nuclear mitochondrial genes that are usually sequenced in a diagnostic setting reached a sequencing depth of more than 30×. Furthermore, an optimal mtDNA genome coverage was achieved for all samples, detecting 100% of mtDNA genome bases covered at 100×, 99% of mtDNA genome bases covered at 1,000× coverage, and 98% of mtDNA genome bases covered at 2,000× as mean values of the 24 batch A samples with 1:50 dilution. Higher dilution molar ratios (1:100 and 1:500) showed a reasonable fall‐off in the mtDNA genome coverage of at least 1,000× to 98% and 18%, respectively. Therefore, a molar ratio of mito:ND baits of 1:50 was used for all Mito‐aND‐Panel analyses.

### 3.2 Variant detection in nuclear genes and mtDNA genome heteroplasmy by Mito‐aND‐Panel

The accuracy and reproducibility of variant detection in the ND‐Panel genes within the Mito‐aND‐Panel were assessed using three reference DNAs (Coriell Institute for Medical Research: NA12889, NA12890, RM8398). Sensitivity and positive predictive value (PPV) for the detection of nuclear single‐nucleotide variants (SNVs) were 99.91% and 99.79% for SNPs and 94% and 97% for InDels respectively, based on 5,623 sites with at least 20× read‐depth, which according to guidelines is sufficient for NGS‐based diagnostic purposes (Matthijs et al., 2016).

The performance of the mitochondrial variant calling of the mtDNA‐Server has been assessed by the authors (Weissensteiner et al., 2016). For that, an analysis with Ion Torrent data with an average coverage of 5,000× showed a sensitivity of 77.78% for heteroplasmy levels of 2% and 81.48% for heteroplasmy levels of 10%. Both specificity and PPV are estimated to be 100% in the tested cases (Weissensteiner et al., 2016). We expect the Mito‐aND‐Panel analysis to outperform these estimations due to a higher average coverage and the lower error rate of Illumina sequencing systems compared to Ion Torrent.

### 3.3 Mito‐aND‐Panel enables a more sensitive heteroplasmy detection compared to Sanger sequencing and whole exome sequencing in leukocytes

To assess the diagnostic yield of the combined nuclear and mitochondrial DNA capturing method, we compared its efficiency to identify mtDNA variants to standard diagnostic procedures using WES and Sanger sequencing/SNaPshot.

A total of 17 positive control samples from patients with known pathogenic variants *MT‐ATP6* (m.8993T > G), *MT‐TK* (m.8344A > G), or *MT‐TL1* (m.3243A > G) in mtDNA were enriched with 1:50 bait dilution and subsequently sequenced by NGS. The level of variant heteroplasmy in this cohort had been previously determined by Sanger sequencing and SNaPshot analysis. In all 17 patients, NGS technology allowed the detection of the underlying homoplasmic or heteroplasmic mtDNA variants.

**Figure 2:**
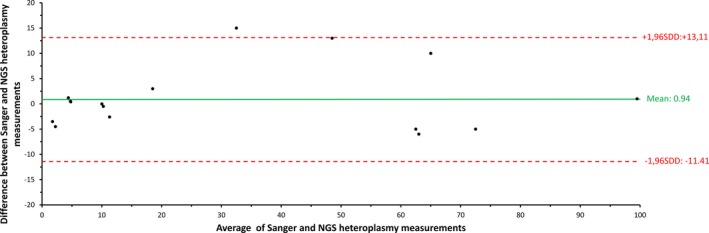
Bland–Altman plot for depicting the agreement between NGS (next‐generation sequencing) and Sanger sequencing/SNaPshot heteroplasmy measurements. On the x‐axis, the average of NGS and Sanger sequencing heteroplasmy is plotted. On the y‐axis, the difference between next‐generation and Sanger sequencing/SNaPshot heteroplasmy is plotted. The mean difference is indicated as green line, the 95% limits of agreement (average difference ± 1.96 standard deviation of the difference) are indicated as red dotted lines. Two of the 17 samples showed exactly the same difference between Sanger sequencing/SNaPshot and NGS heteroplasmy measurements, thus only 15 dots of 17 calculated samples are shown in the figure

A Bland–Altman plot was created for analyzing the agreement between Sanger sequencing and NGS heteroplasmy measurements (Figure 2). The mean value of the difference (NGS/Sanger = 0.94) between the two measurements was different from 0, indicating that there was a systemic difference between the two methods. However, 16 of 17 values lay within the 95% limits of agreement (Figure 2).

Importantly, Mito‐aND‐Panel allowed a more precise detection of heteroplasmic mtDNA variants (up to 3.5%) compared to Sanger sequencing/SNaPshot (Table 1A,B). Interestingly, in one patient, the mtDNA variant *MT‐TL1* (m.3243A > G) was not detected in blood‐extracted DNA by Sanger sequencing/SNaPshot, but had been shown to be present in muscle‐extracted DNA in 70% heteroplasmy. Analysis with Mito‐aND‐Panel was able to detect 3.5% heteroplasmy of the pathogenic mtDNA variant *MT‐TL1* (m.3243A > G) in the blood DNA sample of this patient, indicating a more sensitive variant detection by Mito‐aND‐Panel by a high resolution for low‐heteroplasmy rate due to a high mtDNA coverage of >7,000× compared to the Sanger sequencing/SNaPshot method.

To further assess the efficiency to identify mtDNA variants by Mito‐aND‐Panel compared to WES, 11 blood DNA‐positive control samples were in parallel tested by WES (Table 1B). WES enrichment and sequencing generated a total of nine Megabases of sequence resulting in a mean nuclear exome sequencing depth of 190× with more than 97% of exome targets being covered with at least 30×. Coverage analysis of the mtDNA showed a mean sequencing depth of 39.2 (*SD* 48.0%) and 91.78% of mtDNA genome bases covered at 10×, 60.71% bases covered at 30× coverage, and 2.95% of bases covered at 100×. Heteroplasmy was detectable in seven of 11 patients as WES was not able to detect variants with heteroplasmy levels below 20% (Table 1B).

### 3.4 Detection of NUMTs with the mtDNA‐Server

mtDNA is known to have been inserted into nuclear DNA to form NUMTs over the course of evolution (Mishmar, Ruiz‐Pesini, Brandon, & Wallace, 2004). Since NUMTs share strong sequence similarities with mtDNA genes, off‐target NUMTs capture and the subsequent alignment to the mtDNA genome usually introduces bias in the detection of heteroplasmic mtDNA variants. The mtDNA‐Server annotates variants with known NUMTs according to Dayama et al. (Dayama et al., 2014). We detected both low‐level variants corresponding to known NUMTs and low‐level variants not assigned to known NUMTs regions.

## 4 DISCUSSION

Here, we established the Mito‐aND‐Panel for parallel sequencing of the entire mtDNA and 1,564 nuclear DNA genes, including 308 nuclear mitochondrial disease‐related genes (MitoCarta v2.0) that achieves variant detection at high resolution for low‐heteroplasmic mtDNA mutations (down to 3.5%) at a mean coverage of >7,000×. In addition, analysis of nuclear genes reaches the required accuracy of 99.9% sensitivity in 99% of the analyzed region, as recommended by current guidelines for NGS diagnostics (Matthijs et al., 2016).

Current methods for the genetic diagnosis of inherited mitochondrial diseases had limitations in terms of variant detection accuracy, turnaround time, and costs for separate mtDNA and nuclear DNA analysis. The Mito‐aND‐Panel we present here is a sensitive and accurate method, which overcomes these limitations.

Accurate and sensitive methods are necessary to enable the detection and quantification of low‐heteroplasmic variants of mtDNA. The quantification of mtDNA heteroplasmy is both challenging and of particular clinical relevance because mtDNA mutations exert their phenotypic effect above a certain mutation load threshold, which may vary depending on the type of change and tissue (Chinnery & Hudson, 2013; Gasparre et al., 2011; Rossignol et al., 2003).

A variety of techniques have been used for heteroplasmy detection, including Sanger sequencing (Irwin et al., 2009), SNaPshot (Cassandrini et al., 2006), high‐performance liquid chromatography (HPLC) (Meierhofer, Mayr, Ebner, Sperl, & Kofler, 2005), pyrosequencing (Mikkelsen, Frank‐Hansen, Hansen, & Morling, 2014), high‐resolution melt (HRM) profiling (Dobrowolski et al., 2009), a temporal temperature gradient gel electrophoresis (TTGE) strategy (Wong, Chen, & Tan, 2004), the Invader Assay (Mashima et al., 2004), amplification refractory mutation system (Bai & Wong, 2004), and surveyor nuclease (Bannwarth, Procaccio, & Paquis‐Flucklinger, 2005).

Each of the mentioned strategies has its own disadvantages (Sobenin et al., 2014). For some methods, the candidate heteroplasmic position needs to be defined first, the method may not allow determination of the actual heteroplasmic position, or the method is too labor intensive to be applicable to large numbers of samples. Most of the techniques were unable to detect the mutation when present below 20% and the level of heteroplasmy cannot be quantified accurately. HRM, HPLC, and endonuclease method may be excellent for qualitative detection of heteroplasmy itself, but the methods have insufficient resolution for the quantitative measurement of mtDNA heteroplasmy level. The limit to detect heteroplasmic mtDNA variants in Sanger sequencing may be as low as 6% (Irwin et al., 2009), but in general a detection limit of about 10% to 20% to detect low‐level mutant alleles (Tsiatis et al., 2010) is estimated. SNaPshot sequencing has insufficient resolution in measuring the content of the mutant allele of <5% (Naue et al., 2011) and can only be applied as targeted testing to confirm or to exclude a specific variant*.* Pyrosequencing provides high accuracy for the quantitative measurements of heteroplasmy levels but the method is extremely expensive and may thus limit the possibility of routine analysis of a large number of samples. Moreover, the limit of detection will depend on many factors including sequence quality, personnel experience, sequence context, and use of bidirectional data. Importantly, the efficiency of detection can vary for each method from laboratory to laboratory, as a result of different instruments, chemistries, or standards for calling heteroplasmy (Hancock, Tully, & Levin, 2005; Prieto et al., 2008).

Next‐Generation Sequencing technologies enable studies of heteroplasmy across the entire mtDNA genome at much higher resolution, because many independent reads are generated for each position thus overcoming limitations of current methods. The currently most reliable and sensitive NGS method for detecting almost all clinically relevant mitochondrial DNA defects including both point mutations and large‐scale deletions of mitochondrial DNA is long‐range PCR followed by massive parallel sequencing (Cui et al., 2013). However, this requires several separate reaction steps so that this procedure cannot be directly integrated into the workflow of the standard NGS sequencing of nuclear genes.

The single Mito‐aND enrichment approach we present here was able to generate over 100 Megabases of sequence of mtDNA and two Gigabases of genomic DNA in a single targeted‐capture assay. This translates into >10‐times more fold coverage for mtDNA in relation to nuclear DNA, which is sufficient for accurate heteroplasmic detection and, in addition, allows dual analysis of mtDNA and nuclear genes. High‐coverage NGS sequencing might even detect variants in leukocyte‐extracted DNA which are below the limit of detection in Sanger sequencing, and otherwise have to be identified in different tissues with higher heteroplasmy levels (Whittaker et al., 2009). This is exemplified by the results of one patient who showed 70% heteroplasmy for the MELAS variant m.3243A > G (*MT‐TL1*) in muscle biopsy. In leukocyte‐extracted DNA, the variant was not identified by Sanger sequencing, but Mito‐aND‐Panel was able to detect 3.5% heteroplasmy. Thus, the Mito‐aND‐Panel provides a simple, high‐throughput platform for mitochondrial genome sequencing for accurate mtDNA heteroplasmy detection. However, the method may still miss some low‐level heteroplasmic mtDNA SNVs in blood specimens but high‐level in other type of tissues such as muscle or urine (Whittaker et al., 2009).

Beside point mutations, single large‐scale deletions in mtDNA are a common cause of mitochondrial disease (DiMauro & Hirano, 1993; Picard, Vincent, & Turnbull, 2016). Single large‐scale mtDNA deletions are especially associated with three very well characterized phenotypes, Kearns–Sayre syndrome (KSS), Pearson syndrome, chronic progressive external ophthalmoplegia (CPEO), and rarely Leigh syndrome. Approximately 90% of individuals with KSS have a large‐scale mtDNA deletion that is usually present in all tissues but mutated mtDNA is often undetectable in blood leukocytes, necessitating muscle biopsy. In CPEO, mtDNA deletions are confined to skeletal muscle. In Pearson syndrome, mtDNA deletions are usually more abundant in blood than in other tissues. Currently, a major limitation of the Mito‐aND‐Panel is the missing CNV detection. Even if an adapted bioinformatics pipeline might enable CNV detection, low‐levels of heteroplasmy and mtDNA deletions confined to muscle will remain a limiting factor. Therefore, screening for single large‐scale mtDNA deletions—using a suitable method and tissue—is necessary to complete the diagnosis of mitochondrial disease, especially in the presence of a characteristic clinical phenotype suggesting mtDNA deletion syndrome.

Due to the high sensitivity of NGS sequencing, the detection rate of low‐frequency variants is increasing and interpretation of the data is challenging. Interpretation of mtDNA variants is further complicated by the presence of nuclear DNA regions homologous to mtDNA. These regions, called NUMTs, are the result of an extensive mtDNA pseudogenization in the nuclear genome during evolution. They can be found as blocks of several kilobases, highly homologous to the genuine mtDNA sequence, and spread as multiple copies throughout the genome. Some NUMTs seem to be universal, whereas others may be specific to some subpopulations (Dayama et al., 2014). NGS following targeted enrichment of mtDNA, alone or in combination with targeted enrichment of nuclear DNA, is hampered by the off‐target capture of NUMT sequences. Only amplification of the complete mitochondrial genome using long‐range PCR followed by NGS sequencing provides the greatest level of specific mtDNA enrichment, approaching 100% of the mtDNA avoiding false variant calls in NUMTs, and enabling a successful detection of heteroplasmic variants (Douglas et al., 2014). However, sequencing artefacts arising from the high‐fidelity polymerase diminish variant detection accuracy (Gould et al., 2015) and further analysis of nuclear genes is only possible by an additional separate analysis. Prior separation of the mitochondrial genome from the nuclear genome has been shown to overcome the problems of PCR amplification; however, this method remains nontrivial and is difficult to apply in routine diagnostics (Gould et al., 2015).

We show that our pipeline approach in combination with the mtDNA‐Server workflow, (Weissensteiner et al., 2016), correctly annotated low‐level variants corresponding to known NUMTs. Thus, interpretation of very low‐frequency mtDNA variants is further complicated by NUMTs and the clinical relevance should always be evaluated in context with the ACMG classification, the clinical phenotype of the patient and possibly the detection of the variant in different tissue depending on the tissue‐specific threshold.

Since mitochondrial diseases are very heterogeneous in terms of clinical presentation and the number of possible underlying defects in nuclear‐encoded genes, a common strategy in diagnostics and research is WES. Even without targeted mtDNA sequencing, mtDNA sequences can be extracted from exome sequencing data, greatly increasing the range of mitochondrial genetics data available for research purposes. Various research groups have used this approach to increase their diagnostic yield. Dinwiddie et al. demonstrated the utility of using a standard exome kit for examining the mtDNA and discovery of both homoplasmic and heteroplasmic variants (Dinwiddie et al., 2013). The study presented four cases where exome sequencing yielded a molecular diagnosis after none was identified by conventional methods. One patient was found to have Leigh syndrome due to a mutation in *MT‐ATP6*, two affected siblings were discovered to be compound heterozygous for mutations in the NDUFV1 gene, which causes mitochondrial complex I deficiency, and one patient was found to have coenzyme Q10 deficiency due to compound heterozygous mutations in COQ2. Exome enrichment was conducted with Illumina TruSeq Exome v1 kit (62.2 Megabases). Sequencing was performed on an Illumina HiSeq 2000 using v3 reagents and 1× 101 basepair sequencing reads. Using this method, they obtained greater than 50× coverage of uniquely aligning reads with six Gigabase or more sequencing, which allows for analysis of both the nuclear and mtDNA genomes.

Moreover, Diroma et al. showed that analysis of mitochondrial DNA from off‐target WES enrichment studies is by far not sufficient for a robust detection of low mtDNA heteroplasmies as coverage and sequencing depth are highly variable (between 47% and 97% ≥ 1× coverage and mean mtDNA per base depth between 25 and 410) (Diroma et al., 2014). Our results confirm this observation. We were not able to detect mtDNA pathogenic variants in 4 out of 11 patients with known mtDNA pathogenic heteroplasmic variants from WES data. Mean sequencing depth was between 7.4× and 79.7×, and therefore quite low compared to others (Diroma et al., 2014). This may be due to the used WES NGS technology and coverage might have been higher if using a different sequencing platform. Indeed, several studies reported that there is a difference in the coverage of the off‐target enriched mtDNA using exome kits from different manufactures (Picardi & Pesole, 2012). However, the exome kit and sequencing platform used in our study are widely used and are often applied in clinical diagnostics.

Falk and co‐workers optimized the whole exome analysis by blending additional baits targeting the mtDNA sequence and the complete MitoCarta nuclear gene set into a standard 50 Mb whole exome kit thus optimizing mtDNA coverage (Falk et al., 2012). This approach which showed 99.8% of the mtDNA genome at 1,000× coverage depth enabled heteroplasmy detection between 7.8% and 8.3%. However, 97.5% of the targeted nuclear genomic regions reached a maximum sequencing depth of 10×, which is not sufficient for diagnostic purposes according to current guidelines (Matthijs et al., 2016). Heterozygous variants will be missed in 5% of the cases if 96% of the target is covered at 20×.

Our data show that limiting the analysis to less nuclear‐encoded genes significantly improves the detection rate for mtDNA variants, compared to WES, in addition to a high‐coverage sequencing of nuclear genes after targeted enrichment. Thus, it was possible to meet the diagnostic quality requirements according to international guidelines. The mean coverage of 7,373 for mtDNA sequences corresponded well to the recommended mean coverage of >2,000 for the detection of somatic mutations (Jennings et al., 2017). In all patients, nuclear gene panel analyses fulfilled type B testing according to the EJHG guidelines (Matthijs et al., 2016).

In summary, we demonstrated the custom Agilent SureSelect Mito‐aND‐Panel as an NGS‐based method for the parallel analysis of targeted nuclear genes and the complete mtDNA, which allows accurate detection of low‐level mtDNA heteroplasmy due to a very high sequencing depth, compared to traditional methods in blood samples. Since costs of the custom‐made targeted‐enrichment Mito‐aND‐Panel are lower compared to an entire whole exome or genome sequencing and the method is simple to setup, we anticipate this is a highly relevant cost‐effective method for sequence‐based genetic diagnosis in clinical diagnostic applications. Compared to WES, the limitation of using this panel in suspected nuclear mitochondrial disorders is that pathogenic variants in novel nuclear mitochondrial or nonmitochondrial disease genes are not identified. Thus, for clinical molecular diagnosis the gene list should be updated and the panel should be redesigned regularly. A further technical improvement of targeted enrichment with capture panels, which include all nuclear genes with known human disease association (clinical exome panels), could overcome this limitation in the future.

## CONFLICT OF INTEREST

The authors declare no conflict of interest.
