# Supplementary material for: Mitochondrial and nuclear disease panel (Mito‐aND‐Panel): Combined sequencing of mitochondrial and nuclear DNA by a cost‐effective and sensitive NGS‐based method
Source: Mol Genet Genomic Med. 2018 Nov 8;6(6):1188–98. doi: 10.1002/mgg3.500 (PMC6305657; doi:10.1002/mgg3.500)
Supplement: Supplementary file 1 [file MGG3-6-1188-s001.docx]

**Suppl. Table 1:** List of genes analysed by the Mito-aND-Panel. Genes from MitoCarta database are marked in green.

| **Gene Name** | **OMIM** | **RefSeq** | **Gene Name** | **OMIM** | **RefSeq** | **Gene Name** | **OMIM** | **RefSeq** | **Gene Name** | **OMIM** | **RefSeq** |
| --- | --- | --- | --- | --- | --- | --- | --- | --- | --- | --- | --- |
| A2ML1 | 610627 | NM_144670.5 | DLD | 238331 | NM_000108.4 | LMNA | 150330 | NM_005572.3 | ROR2 | 602337 | NM_004560.3 |
| AAAS | 605378 | NM_015665.5 | DLG3 | 300189 | NM_021120.3 | LMNB1 | 150340 | NM_005573.3 | RPL10 | 312173 | NM_006013.4 |
| AARS | 601065 | NM_001605.2 | DLL1 | 606582 | NM_005618.3 | LMNB2 | 150341 | NM_032737.3 | RPS6KA3 | 300075 | NM_004586.2 |
| AARS2 | 612035 | NM_020745.3 | DLL4 | 605185 | NM_019074.3 | LMOD3 | 616112 | NM_001304418.1 | RRAS | 165090 | NM_006270.4 |
| ABCA1 | 600046 | NM_005502.3 | DMD | 300377 | NM_004006.2 | LMX1B | 602575 | NM_002316.3 | RRM2B | 604712 | NM_001172477.1 |
| ABCB11 | 603201 | NM_003742.2 | DNA2 | 601810 | NM_001080449.2 | LONP1 | 605490 | NM_001276479.1 | RSPH4A | 612647 | NM_001010892.2 |
| ABCB4 | 171060 | NM_000443.3 | DNAAF1 | 613190 | NM_178452.5 | LPIN1 | 605518 | NM_145693.2 | RSPH9 | 612648 | NM_152732.4 |
| ABCB6 | 605452 | NM_005689.2 | DNAAF2 | 612518 | NM_018139.2 | LRIG2 | 608869 | NM_014813.2 | RTN2 | 603183 | NM_005619.4 |
| ABCB7 | 300135 | NM_004299.4 | DNAAF3 | 614566 | NM_001256714.1 | LRP2 | 600073 | NM_004525.2 | RUBCN | 613516 | NM_001145642.3 |
| ABCC2 | 601107 | NM_000392.4 | DNAH11 | 603339 | NM_001277115.1 | LRP4 | 604270 | NM_002334.3 | RYR1 | 180901 | NM_000540.2 |
| ABCC6 | 603234 | NM_001171.5 | DNAH5 | 608644 | NM_001369.2 | LRP5 | 603506 | NM_002335.3 | RYR2 | 180902 | NM_001035.2 |
| ABCC8 | 600509 | NM_000352.4 | DNAI1 | 604366 | NM_012144.3 | LRPPRC | 607544 | NM_133259.3 | SACS | 604490 | NM_014363.5 |
| ABCC9 | 601439 | NM_020297.3 | DNAI2 | 605483 | NM_023036.4 | LRRK2 | 609007 | NM_198578.3 | SALL1 | 602218 | NM_002968.2 |
| ABCD1 | 300371 | NM_000033.3 | DNAJB2 | 604139 | NM_001039550.1 | LRSAM1 | 610933 | NM_138361.5 | SALL2 | 602219 | NM_005407.2 |
| ABHD12 | 613599 | NM_001042472.2 | DNAJB5 | 611328 | NM_001135004.2 | LTBP2 | 602091 | NM_000428.2 | SALL4 | 607343 | NM_020436.4 |
| ABHD5 | 604780 | NM_016006.4 | DNAJB6 | 611332 | NM_058246.3 | LYRM7 | 615831 | NM_181705.3 | SAMHD1 | 606754 | NM_015474.3 |
| ACAD9 | 611103 | NM_014049.4 | DNAJC19 | 608977 | NM_145261.3 | LYST | 606897 | NM_000081.3 | SARS2 | 612804 | NM_017827.3 |
| ACADM | 607008 | NM_000016.5 | DNAJC3 | 601184 | NM_006260.4 | LZTR1 | 600574 | NM_006767.3 | SBDS | 607444 | NM_016038.2 |
| ACADS | 606885 | NM_000017.3 | DNAJC5 | 611203 | NM_025219.2 | MAB21L2 | 604357 | NM_006439.4 | SBF1 | 603560 | NM_002972.3 |
| ACADVL | 609575 | NM_000018.3 | DNM1L | 603850 | NM_012062.4 | MAF | 177075 | NM_005360.4 | SBF2 | 607697 | NM_030962.3 |
| ACE | 106180 | NM_000789.3 | DNM2 | 602378 | NM_001005360.2 | MAG | 159460 | NM_080600.2 | SCARB2 | 602257 | NM_005506.3 |
| ACO2 | 100850 | NM_001098.2 | DNMT1 | 126375 | NM_001130823.2 | MAGEL2 | 605283 | NM_019066.4 | SCARF2 | 613619 | NM_153334.6 |
| ACOX1 | 609751 | NM_004035.6 | DNMT3A | 602769 | NM_022552.4 | MAGT1 | 300715 | NM_032121.5 | SCN10A | 604427 | NM_006514.3 |
| ACSL4 | 300157 | NM_004458.2 | DNMT3B | 602900 | NM_006892.3 | MAN1B1 | 604346 | NM_016219.4 | SCN11A | 604385 | NM_014139.2 |
| ACTA1 | 102610 | NM_001100.3 | DOCK6 | 614194 | NM_020812.3 | MAN2B1 | 609458 | NM_000528.3 | SCN1A | 182389 | NM_006920.4 |
| ACTA2 | 102620 | NM_001613.2 | DOCK7 | 615730 | NM_033407.3 | MANBA | 609489 | NM_005908.3 | SCN1B | 600235 | NM_001037.4 |
| ACTB | 102630 | NM_001101.3 | DOK7 | 610285 | NM_173660.4 | MAOA | 309850 | NM_000240.3 | SCN2A | 182390 | NM_021007.2 |
| ACTC1 | 102540 | NM_005159.4 | DOLK | 610746 | NM_014908.3 | MAP2K1 | 176872 | NM_002755.3 | SCN4A | 603967 | NM_000334.4 |
| ACTG1 | 102560 | NM_001614.3 | DPAGT1 | 191350 | NM_001382.3 | MAP2K2 | 601263 | NM_030662.3 | SCN4B | 608256 | NM_174934.3 |
| ACTG2 | 102545 | NM_001615.3 | DPM1 | 603503 | NM_003859.2 | MAPT | 157140 | NM_005910.5 | SCN5A | 600163 | NM_198056.2 |
| ACTN2 | 102573 | NM_001103.3 | DPM2 | 603564 | NM_003863.3 | MARS | 156560 | NM_004990.3 | SCN8A | 600702 | NM_014191.3 |
| ACTN4 | 604638 | NM_004924.5 | DPM3 | 605951 | NM_153741.1 | MARS2 | 609728 | NM_138395.3 | SCN9A | 603415 | NM_002977.3 |
| ACVRL1 | 601284 | NM_000020.2 | DSC2 | 125645 | NM_004949.4 | MAT2A | 601468 | NM_005911.5 | SCO1 | 603644 | NM_004589.3 |
| ACY1 | 104620 | NM_000666.2 | DSG2 | 125671 | NM_001943.4 | MATR3 | 164015 | NM_199189.2 | SCO2 | 604272 | NM_005138.2 |
| ADAMTS10 | 608990 | NM_030957.3 | DSP | 125647 | NM_004415.3 | MBD5 | 611472 | NM_018328.4 | SDHA | 600857 | NM_004168.3 |
| ADAMTS17 | 607511 | NM_139057.3 | DST | 113810 | NM_001723.5 | MBTPS2 | 300294 | NM_015884.3 | SDHAF1 | 612848 | NM_001042631.2 |
| ADAMTS2 | 604539 | NM_014244.4 | DSTYK | 612666 | NM_015375.2 | MCEE | 608419 | NM_032601.3 | SDHAF2 | 613019 | NM_017841.2 |
| ADAMTSL4 | 610113 | NM_019032.5 | DVL1 | 601365 | NM_004421.2 | MCOLN1 | 605248 | NM_020533.2 | SDHB | 185470 | NM_003000.2 |
| ADAR | 146920 | NM_001111.4 | DYNC1H1 | 600112 | NM_001376.4 | MCPH1 | 607117 | NM_024596.4 | SDHC | 602413 | NM_003001.3 |
| ADCK3 | 606980 | NM_020247.4 | DYNC2H1 | 603297 | NM_001080463.1 | MECP2 | 300005 | NM_004992.3 | SDHD | 602690 | NM_003002.3 |
| ADCK4 | 615567 | NM_024876.3 | DYRK1A | 600855 | NM_001396.3 | MED12 | 300188 | NM_005120.2 | SEC63 | 608648 | NM_007214.4 |
| ADCY5 | 600293 | NM_183357.2 | DYSF | 603009 | NM_003494.3 | MED13L | 608771 | NM_015335.4 | SECISBP2 | 607693 | NM_024077.4 |
| ADCY6 | 600294 | NM_015270.4 | DYX1C1 | 608706 | NM_130810.3 | MED23 | 605042 | NM_015979.3 | SEPN1 | 606210 | NM_020451.2 |
| ADGRG1 | 604110 | NM_005682.6 | EARS2 | 612799 | NM_001083614.1 | MED25 | 610197 | NM_030973.3 | SEPSECS | 613009 | NM_016955.3 |
| ADGRG6 | 612243 | NM_020455.5 | ECEL1 | 605896 | NM_004826.3 | MEF2C | 600662 | NM_002397.4 | SEPT9 | 604061 | NM_006640.4 |
| ADSL | 608222 | NM_000026.3 | ECHS1 | 602292 | NM_004092.3 | MEGF10 | 612453 | NM_032446.2 | SERAC1 | 614725 | NM_032861.3 |
| ADSSL1 | 612498 | NM_152328.4 | EDN3 | 131242 | NM_207034.2 | MFAP5 | 601103 | NM_003480.3 | SERPINA1 | 107400 | NM_000295.4 |
| AFF2 | 300806 | NM_002025.3 | EDNRA | 131243 | NM_001957.3 | MFN2 | 608507 | NM_014874.3 | SETBP1 | 611060 | NM_015559.2 |
| AFG3L2 | 604581 | NM_006796.2 | EDNRB | 131244 | NM_000115.3 | MFRP | 606227 | NM_031433.3 | SETD2 | 612778 | NM_014159.6 |
| AGBL1 | 615496 | NM_152336.2 | EEF1A2 | 602959 | NM_001958.3 | MFSD8 | 611124 | NM_152778.2 | SETD5 | 615743 | NM_001292043.1 |
| AGK | 610345 | NM_018238.3 | EEF2 | 130610 | NM_001961.3 | MGAT2 | 602616 | NM_002408.3 | SETX | 608465 | NM_015046.5 |
| AGL | 610860 | NM_000642.2 | EFEMP2 | 604633 | NM_016938.4 | MGME1 | 615076 | NM_052865.3 | SF3B4 | 605593 | NM_005850.4 |
| AGRN | 103320 | NM_198576.3 | EFHC1 | 608815 | NM_018100.3 | MICAL1 | 607129 | NM_001286613.1 | SGCA | 600119 | NM_000023.3 |
| AGT | 106150 | NM_000029.3 | EFNB1 | 300035 | NM_004429.4 | MICU1 | 605084 | NM_006077.3 | SGCB | 600900 | NM_000232.4 |
| AGTR1 | 106165 | NM_031850.3 | EFTUD2 | 603892 | NM_004247.3 | MID1 | 300552 | NM_000381.3 | SGCD | 601411 | NM_000337.5 |
| AGTR2 | 300034 | NM_000686.4 | EGR2 | 129010 | NM_000399.4 | MIP | 154050 | NM_012064.3 | SGCE | 604149 | NM_003919.2 |
| AGXT | 604285 | NM_000030.2 | EHMT1 | 607001 | NM_024757.4 | MIR184 | 613146 | NR_029705.1 | SGCG | 608896 | NM_000231.2 |
| AIFM1 | 300169 | NM_004208.3 | EIF2AK3 | 604032 | NM_004836.6 | MITF | 156845 | NM_000248.3 | SGSH | 605270 | NM_000199.3 |
| AIMP1 | 603605 | NM_004757.3 | EIF2AK4 | 609280 | NM_001013703.3 | MKS1 | 609883 | NM_017777.3 | SH3BP4 | 605611 | NM_014521.2 |
| AKAP9 | 604001 | NM_005751.4 | EIF2B1 | 606686 | NM_001414.3 | MLC1 | 605908 | NM_015166.3 | SH3TC2 | 608206 | NM_024577.3 |
| AKR1D1 | 604741 | NM_005989.3 | EIF2B2 | 606454 | NM_014239.3 | MMAA | 607481 | NM_172250.2 | SHANK3 | 606230 | NM_033517.1 |
| AKT1 | 164730 | NM_001014432.1 | EIF2B3 | 606273 | NM_020365.4 | MMAB | 607568 | NM_052845.3 | SHH | 600725 | NM_000193.3 |
| AKT3 | 611223 | NM_005465.4 | EIF2B4 | 606687 | NM_015636.3 | MMACHC | 609831 | NM_015506.2 | SHOC2 | 602775 | NM_007373.3 |
| ALAD | 125270 | NM_000031.5 | EIF2B5 | 603945 | NM_003907.2 | MOCS1 | 603707 | NM_005943.5 | SHROOM4 | 300579 | NM_020717.3 |
| ALAS2 | 301300 | NM_000032.4 | EIF2S3 | 300161 | NM_001415.3 | MOCS2 | 603708 | NM_176806.3 | SIGMAR1 | 601978 | NM_005866.3 |
| ALDH18A1 | 138250 | NM_002860.3 | ELN | 130160 | NM_001081755.2 | MOGS | 601336 | NM_006302.2 | SIK1 | 605705 | NM_173354.3 |
| ALDH1A3 | 600463 | NM_000693.3 | ELOVL4 | 605512 | NM_022726.3 | MORC2 | 616661 | NM_014941.3 | SIL1 | 608005 | NM_022464.4 |
| ALDH3A2 | 609523 | NM_000382.2 | EMD | 300384 | NM_000117.2 | MPDU1 | 604041 | NM_004870.3 | SIPA1L3 | 616655 | NM_015073.2 |
| ALDH7A1 | 107323 | NM_001182.4 | EMP2 | 602334 | NM_001424.5 | MPI | 154550 | NM_002435.2 | SIX1 | 601205 | NM_005982.3 |
| ALG1 | 605907 | NM_019109.4 | EMX2 | 600035 | NM_004098.3 | MPV17 | 137960 | NM_002437.4 | SIX2 | 604994 | NM_016932.4 |
| ALG11 | 613666 | NM_001004127.2 | ENG | 131195 | NM_000118.3 | MPZ | 159440 | NM_000530.7 | SIX3 | 603714 | NM_005413.3 |
| ALG12 | 607144 | NM_024105.3 | ENTPD1 | 601752 | NM_001776.5 | MR1 | 600764 | NM_001194999.1 | SIX5 | 600963 | NM_175875.4 |
| ALG13 | 300776 | NM_001099922.2 | EOGT | 614789 | NM_001278689.1 | MRE11A | 600814 | NM_005591.3 | SIX6 | 606326 | NM_007374.2 |
| ALG14 | 612866 | NM_144988.3 | EOMES | 604615 | NM_005442.3 | MRPL44 | 611849 | NM_022915.3 | SKI | 164780 | NM_003036.3 |
| ALG2 | 607905 | NM_033087.3 | EP300 | 602700 | NM_001429.3 | MRPS16 | 609204 | NM_016065.3 | SLC12A1 | 600839 | NM_000338.2 |
| ALG3 | 608750 | NM_005787.5 | EPB41L1 | 602879 | NM_012156.2 | MRPS22 | 605810 | NM_020191.2 | SLC12A3 | 600968 | NM_000339.2 |
| ALG6 | 604566 | NM_013339.3 | EPG5 | 615068 | NM_020964.2 | MRPS7 | 611974 | NM_015971.3 | SLC12A6 | 604878 | NM_005135.2 |
| ALG8 | 608103 | NM_024079.4 | EPHA2 | 176946 | NM_004431.3 | MTFMT | 611766 | NM_139242.3 | SLC13A5 | 608305 | NM_177550.4 |
| ALG9 | 606941 | NM_024740.2 | EPHX1 | 132810 | NM_000120.3 | MTHFR | 607093 | NM_005957.4 | SLC16A12 | 611910 | NM_213606.3 |
| ALPL | 171760 | NM_000478.5 | EPM2A | 607566 | NM_005670.3 | MTM1 | 300415 | NM_000252.2 | SLC16A2 | 300095 | NM_006517.4 |
| ALS2 | 606352 | NM_020919.3 | ERBB3 | 190151 | NM_001982.3 | MTMR1 | 300171 | NM_003828.3 | SLC17A5 | 604322 | NM_012434.4 |
| ALX1 | 601527 | NM_006982.2 | ERCC1 | 126380 | NM_202001.2 | MTMR14 | 611089 | NM_022485.4 | SLC19A2 | 603941 | NM_006996.2 |
| ALX3 | 606014 | NM_006492.2 | ERCC2 | 126340 | NM_000400.3 | MTMR2 | 603557 | NM_016156.5 | SLC19A3 | 606152 | NM_025243.3 |
| ALX4 | 605420 | NM_021926.3 | ERCC5 | 133530 | NM_000123.3 | MTO1 | 614667 | NM_012123.3 | SLC1A3 | 600111 | NM_004172.4 |
| AMACR | 604489 | NM_014324.5 | ERCC6 | 609413 | NM_000124.3 | MTPAP | 613669 | NM_018109.3 | SLC1A4 | 600229 | NM_003038.4 |
| AMPD1 | 102770 | NM_000036.2 | ERCC8 | 609412 | NM_000082.3 | MTTP | 157147 | NM_000253.3 | SLC22A5 | 603377 | NM_003060.3 |
| AMPD2 | 102771 | NM_001257360.1 | ERLIN1 | 611604 | NM_001100626.1 | MUC1 | 158340 | NM_002456.5 | SLC24A5 | 609802 | NM_205850.2 |
| AMT | 238310 | NM_000481.3 | ERLIN2 | 611605 | NM_007175.6 | MUSK | 601296 | NM_005592.3 | SLC25A1 | 190315 | NM_005984.4 |
| ANG | 105850 | NM_001145.4 | ETFA | 608053 | NM_000126.3 | MUT | 609058 | NM_000255.3 | SLC25A12 | 603667 | NM_003705.4 |
| ANK2 | 106410 | NM_001148.4 | ETFB | 130410 | NM_001985.2 | MYBPC1 | 160794 | NM_002465.3 | SLC25A13 | 603859 | NM_014251.2 |
| ANK3 | 600465 | NM_020987.4 | ETFDH | 231675 | NM_004453.3 | MYBPC3 | 600958 | NM_000256.3 | SLC25A15 | 603861 | NM_014252.3 |
| ANKRD1 | 609599 | NM_014391.2 | ETHE1 | 608451 | NM_014297.4 | MYF6 | 159991 | NM_002469.2 | SLC25A19 | 606521 | NM_021734.4 |
| ANKRD11 | 611192 | NM_013275.5 | ETV5 | 601600 | NM_004454.2 | MYH11 | 160745 | NM_002474.2 | SLC25A20 | 613698 | NM_000387.5 |
| ANLN | 616027 | NM_018685.4 | EXOSC3 | 606489 | NM_016042.3 | MYH14 | 608568 | NM_024729.3 | SLC25A22 | 609302 | NM_024698.5 |
| ANO10 | 613726 | NM_018075.3 | EXOSC8 | 606019 | NM_181503.2 | MYH2 | 160740 | NM_017534.5 | SLC25A26 | 611037 | NM_173471.3 |
| ANO3 | 610110 | NM_031418.3 | EYA1 | 601653 | NM_000503.5 | MYH3 | 160720 | NM_002470.3 | SLC25A3 | 600370 | NM_005888.3 |
| ANO5 | 608662 | NM_213599.2 | EZH2 | 601573 | NM_004456.4 | MYH6 | 160710 | NM_002471.3 | SLC25A38 | 610819 | NM_017875.2 |
| ANOS1 | 300836 | NM_000216.3 | FA2H | 611026 | NM_024306.4 | MYH7 | 160760 | NM_000257.3 | SLC25A4 | 103220 | NM_001151.3 |
| AP1S2 | 300629 | NM_003916.4 | FAH | 613871 | NM_000137.2 | MYH8 | 160741 | NM_002472.2 | SLC25A42 | 610823 | NM_001321544.1 |
| AP4B1 | 607245 | NM_006594.4 | FAM126A | 610531 | NM_032581.3 | MYH9 | 160775 | NM_002473.5 | SLC25A46 | 610826 | NM_001303250.1 |
| AP4E1 | 607244 | NM_007347.4 | FAM134B | 613114 | NM_001034850.2 | MYL2 | 160781 | NM_000432.3 | SLC27A5 | 603314 | NM_012254.2 |
| AP4M1 | 602296 | NM_004722.3 | FAM20C | 611061 | NM_020223.3 | MYL3 | 160790 | NM_000258.2 | SLC2A1 | 138140 | NM_006516.2 |
| AP4S1 | 607243 | NM_007077.4 | FAM58A | 300708 | NM_152274.4 | MYLK | 600922 | NM_053025.3 | SLC2A10 | 606145 | NM_030777.3 |
| AP5Z1 | 613653 | NM_014855.2 | FANCB | 300515 | NM_001018113.2 | MYO18B | 607295 | NM_032608.6 | SLC30A10 | 611146 | NM_018713.2 |
| APOB | 107730 | NM_000384.2 | FARS2 | 611592 | NM_006567.4 | MYO1E | 601479 | NM_004998.3 | SLC33A1 | 603690 | NM_004733.3 |
| APOE | 107741 | NM_000041.3 | FASTKD2 | 612322 | NM_014929.3 | MYO5B | 606540 | NM_001080467.2 | SLC35A1 | 605634 | NM_006416.4 |
| APOL1 | 603743 | NM_145343.2 | FAT1 | 600976 | NM_005245.3 | MYO9A | 604875 | NM_006901.3 | SLC35A2 | 314375 | NM_001042498.2 |
| APOPT1 | 616003 | NM_001302653.1 | FAT4 | 612411 | NM_024582.4 | MYOC | 601652 | NM_000261.1 | SLC35A3 | 605632 | NM_012243.2 |
| APP | 104760 | NM_000484.3 | FBLN5 | 604580 | NM_006329.3 | MYOD1 | 159970 | NM_002478.4 | SLC35C1 | 605881 | NM_018389.4 |
| APPL1 | 604299 | NM_012096.2 | FBN1 | 134797 | NM_000138.4 | MYOT | 604103 | NM_006790.2 | SLC37A4 | 602671 | NM_001164277.1 |
| APTX | 606350 | NM_175073.2 | FBN2 | 612570 | NM_001999.3 | MYOZ2 | 605602 | NM_016599.4 | SLC38A8 | 615585 | NM_001080442.2 |
| ARFGEF2 | 605371 | NM_006420.2 | FBXL4 | 605654 | NM_012160.4 | NAA10 | 300013 | NM_003491.3 | SLC39A13 | 608735 | NM_152264.4 |
| ARG1 | 608313 | NM_000045.3 | FBXO38 | 608533 | NM_030793.4 | NAGLU | 609701 | NM_000263.3 | SLC3A1 | 104614 | NM_000341.3 |
| ARHGAP31 | 610911 | NM_020754.3 | FBXO7 | 605648 | NM_012179.3 | NALCN | 611549 | NM_052867.2 | SLC45A2 | 606202 | NM_016180.4 |
| ARHGDIA | 601925 | NM_001301241.1 | FDX2 | 614585 | NM_001031734.3 | NARS2 | 612803 | NM_024678.5 | SLC4A11 | 610206 | NM_032034.3 |
| ARHGEF10 | 608136 | NM_014629.3 | FECH | 612386 | NM_000140.3 | NBAS | 608025 | NM_015909.3 | SLC52A2 | 607882 | NM_024531.4 |
| ARHGEF15 | 608504 | NM_173728.3 | FGD1 | 300546 | NM_004463.2 | NDE1 | 609449 | NM_001143979.1 | SLC52A3 | 613350 | NM_033409.3 |
| ARHGEF6 | 300267 | NM_004840.2 | FGD4 | 611104 | NM_139241.3 | NDP | 300658 | NM_000266.3 | SLC5A7 | 608761 | NM_021815.4 |
| ARHGEF9 | 300429 | NM_015185.2 | FGF14 | 601515 | NM_004115.3 | NDRG1 | 605262 | NM_006096.3 | SLC6A19 | 608893 | NM_001003841.2 |
| ARID1A | 603024 | NM_006015.4 | FGF20 | 605558 | NM_019851.2 | NDUFA1 | 300078 | NM_004541.3 | SLC6A3 | 126455 | NM_001044.4 |
| ARID1B | 614556 | NM_020732.3 | FGF8 | 600483 | NM_033163.3 | NDUFA10 | 603835 | NM_004544.3 | SLC6A5 | 604159 | NM_004211.4 |
| ARL6IP1 | 607669 | NM_015161.2 | FGFR1 | 136350 | NM_023110.2 | NDUFA11 | 612638 | NM_175614.4 | SLC6A8 | 300036 | NM_005629.3 |
| ARSA | 607574 | NM_000487.5 | FGFR2 | 176943 | NM_000141.4 | NDUFA12 | 614530 | NM_018838.4 | SLC7A9 | 604144 | NM_014270.4 |
| ARSI | 610009 | NM_001012301.2 | FGFR3 | 134934 | NM_000142.4 | NDUFA13 | 609435 | NM_015965.6 | SLC9A6 | 300231 | NM_006359.2 |
| ARX | 300382 | NM_139058.2 | FHL1 | 300163 | NM_001449.4 | NDUFA2 | 602137 | NM_002488.4 | SLCO1B1 | 604843 | NM_006446.4 |
| ASAH1 | 613468 | NM_177924.4 | FIG4 | 609390 | NM_014845.5 | NDUFA9 | 603834 | NM_005002.4 | SLCO1B3 | 605495 | NM_019844.3 |
| ASCC1 | 614215 | NM_001198800.2 | FKBP10 | 607063 | NM_021939.3 | NDUFAF1 | 606934 | NM_016013.3 | SMAD3 | 603109 | NM_005902.3 |
| ASL | 608310 | NM_000048.3 | FKBP14 | 614505 | NM_017946.3 | NDUFAF2 | 609653 | NM_174889.4 | SMAD6 | 602931 | NM_005585.4 |
| ASPA | 608034 | NM_000049.2 | FKRP | 606596 | NM_024301.4 | NDUFAF3 | 612911 | NM_199069.1 | SMAD9 | 603295 | NM_001127217.2 |
| ASPH | 600582 | NM_004318.3 | FKTN | 607440 | NM_001079802.1 | NDUFAF4 | 611776 | NM_014165.3 | SMARCA2 | 600014 | NM_003070.4 |
| ASPM | 605481 | NM_018136.4 | FLAD1 | 610595 | NM_025207.4 | NDUFAF5 | 612360 | NM_024120.4 | SMARCA4 | 603254 | NM_001128849.1 |
| ASS1 | 603470 | NM_000050.4 | FLNA | 300017 | NM_001456.3 | NDUFAF6 | 612392 | NM_152416.3 | SMARCAL1 | 606622 | NM_014140.3 |
| ATL1 | 606439 | NM_015915.4 | FLNB | 603381 | NM_001457.3 | NDUFB11 | 300403 | NM_001135998.2 | SMARCB1 | 601607 | NM_003073.4 |
| ATL3 | 609369 | NM_015459.4 | FLNC | 102565 | NM_001458.4 | NDUFB3 | 603839 | NM_002491.2 | SMARCE1 | 603111 | NM_003079.4 |
| ATM | 607585 | NM_000051.3 | FLRT1 | 604806 | NM_013280.4 | NDUFB9 | 601445 | NM_005005.2 | SMC1A | 300040 | NM_001281463.1 |
| ATOH7 | 609875 | NM_145178.3 | FLVCR2 | 610865 | NM_017791.2 | NDUFS1 | 157655 | NM_005006.6 | SMC3 | 606062 | NM_005445.3 |
| ATP13A2 | 610513 | NM_022089.3 | FMR1 | 309550 | NM_002024.5 | NDUFS2 | 602985 | NM_004550.4 | SMCHD1 | 614982 | NM_015295.2 |
| ATP1A2 | 182340 | NM_000702.3 | FOLR1 | 136430 | NM_016725.2 | NDUFS3 | 603846 | NM_004551.2 | SMN1 | 600354 | NM_000344.3 |
| ATP1A3 | 182350 | NM_152296.4 | FOXC1 | 601090 | NM_001453.2 | NDUFS4 | 602694 | NM_002495.3 | SMOC1 | 608488 | NM_001034852.2 |
| ATP2A1 | 108730 | NM_173201.3 | FOXE3 | 601094 | NM_012186.2 | NDUFS6 | 603848 | NM_004553.4 | SMPD1 | 607608 | NM_000543.4 |
| ATP2B4 | 108732 | NM_001001396.2 | FOXG1 | 164874 | NM_005249.4 | NDUFS7 | 601825 | NM_024407.4 | SMS | 300105 | NM_004595.4 |
| ATP5A1 | 164360 | NM_001001937.1 | FOXH1 | 603621 | NM_003923.2 | NDUFS8 | 602141 | NM_002496.3 | SNAI2 | 602150 | NM_003068.4 |
| ATP5E | 606153 | NM_006886.3 | FOXL2 | 605597 | NM_023067.3 | NDUFV1 | 161015 | NM_007103.3 | SNAP25 | 600322 | NM_003081.4 |
| ATP6AP2 | 300556 | NM_005765.2 | FOXP1 | 605515 | NM_032682.5 | NDUFV2 | 600532 | NM_021074.4 | SNCA | 163890 | NM_000345.3 |
| ATP7A | 300011 | NM_000052.6 | FOXP2 | 605317 | NM_014491.3 | NEB | 161650 | NM_001271208.1 | SNTA1 | 601017 | NM_003098.2 |
| ATP7B | 606882 | NM_000053.3 | FOXP3 | 300292 | NM_014009.3 | NEFL | 162280 | NM_006158.4 | SNX14 | 616105 | NM_001304479.1 |
| ATP8A2 | 605870 | NM_016529.5 | FOXRED1 | 613622 | NM_017547.3 | NEK9 | 609798 | NM_033116.4 | SOD1 | 147450 | NM_000454.4 |
| ATP8B1 | 602397 | NM_005603.4 | FRAS1 | 607830 | NM_025074.6 | NEU1 | 608272 | NM_000434.3 | SOS1 | 182530 | NM_005633.3 |
| ATPAF2 | 608918 | NM_145691.3 | FREM1 | 608944 | NM_144966.5 | NEUROD1 | 601724 | NM_002500.4 | SOS2 | 601247 | NM_006939.2 |
| ATRX | 300032 | NM_000489.4 | FREM2 | 608945 | NM_207361.5 | NEXN | 613121 | NM_144573.3 | SOX10 | 602229 | NM_006941.3 |
| AUH | 600529 | NM_001698.2 | FRMD7 | 300628 | NM_194277.2 | NF1 | 613113 | NM_000267.3 | SOX17 | 610928 | NM_022454.3 |
| B3GALNT2 | 610194 | NM_152490.4 | FTL | 134790 | NM_000146.3 | NFATC2IP | 614525 | NM_032815.3 | SOX2 | 184429 | NM_003106.3 |
| B3GALT6 | 615291 | NM_080605.3 | FTSJ1 | 300499 | NM_012280.3 | NFIX | 164005 | NM_002501.3 | SOX3 | 313430 | NM_005634.2 |
| B3GLCT | 610308 | NM_194318.3 | FUCA1 | 612280 | NM_000147.4 | NFU1 | 608100 | NM_001002755.2 | SPAST | 604277 | NM_014946.3 |
| B4GALNT1 | 601873 | NM_001478.4 | FUS | 137070 | NM_004960.3 | NGF | 162030 | NM_002506.2 | SPATA5 | 613940 | NM_145207.2 |
| B4GALT1 | 137060 | NM_001497.3 | FXN | 606829 | NM_000144.4 | NGLY1 | 610661 | NM_001145294.1 | SPECC1L | 614140 | NM_015330.4 |
| B4GALT7 | 604327 | NM_007255.2 | FYCO1 | 607182 | NM_024513.3 | NHLRC1 | 608072 | NM_198586.2 | SPEG | 615950 | NM_005876.4 |
| B4GAT1 | 605517 | NM_006876.2 | FZD4 | 604579 | NM_012193.3 | NHS | 300457 | NM_198270.3 | SPG11 | 610844 | NM_025137.3 |
| BAAT | 602938 | NM_001701.3 | G6PC | 613742 | NM_000151.3 | NIPA1 | 608145 | NM_144599.4 | SPG20 | 607111 | NM_015087.4 |
| BAG3 | 603883 | NM_004281.3 | GAA | 606800 | NM_000152.4 | NIPBL | 608667 | NM_133433.3 | SPG21 | 608181 | NM_016630.6 |
| BCKDHA | 608348 | NM_000709.3 | GABRA1 | 137160 | NM_000806.5 | NKX2-1 | 600635 | NM_001079668.2 | SPG7 | 602783 | NM_003119.3 |
| BCKDHB | 248611 | NM_183050.3 | GABRD | 137163 | NM_000815.4 | NKX2-5 | 600584 | NM_004387.3 | SPR | 182125 | NM_003124.4 |
| BCL11A | 606557 | NM_022893.3 | GABRG2 | 137164 | NM_000816.3 | NKX2-6 | 611770 | NM_001136271.2 | SPRED1 | 609291 | NM_152594.2 |
| BCOR | 300485 | NM_001123383.1 | GAD1 | 605363 | NM_000817.2 | NLGN3 | 300336 | NM_018977.3 | SPTAN1 | 182810 | NM_001130438.2 |
| BCS1L | 603647 | NM_004328.4 | GALC | 606890 | NM_000153.3 | NLGN4X | 300427 | NM_020742.3 | SPTBN2 | 604985 | NM_006946.2 |
| BFSP1 | 603307 | NM_001195.4 | GALK1 | 604313 | NM_000154.1 | NLRP3 | 606416 | NM_004895.4 | SPTLC1 | 605712 | NM_006415.3 |
| BFSP2 | 603212 | NM_003571.3 | GALNS | 612222 | NM_000512.4 | NME8 | 607421 | NM_016616.4 | SPTLC2 | 605713 | NM_004863.3 |
| BICC1 | 614295 | NM_001080512.2 | GALT | 606999 | NM_000155.3 | NOD2 | 605956 | NM_022162.2 | SPTLC3 | 611120 | NM_018327.2 |
| BICD2 | 609797 | NM_001003800.1 | GAMT | 601240 | NM_000156.5 | NODAL | 601265 | NM_018055.4 | SQSTM1 | 601530 | NM_003900.4 |
| BIN1 | 601248 | NM_139343.2 | GAN | 605379 | NM_022041.3 | NOG | 602991 | NM_005450.4 | SRCAP | 611421 | NM_006662.2 |
| BLK | 191305 | NM_001715.2 | GARS | 600287 | NM_002047.3 | NOL3 | 605235 | NM_001276312.1 | SRD5A3 | 611715 | NM_024592.4 |
| BMP4 | 112262 | NM_001202.4 | GAS1 | 139185 | NM_002048.2 | NOP56 | 614154 | NM_006392.3 | SRPK3 | 301002 | NM_001170761.1 |
| BMP7 | 112267 | NM_001719.2 | GATA3 | 131320 | NM_001002295.1 | NOTCH1 | 190198 | NM_017617.4 | SRPX2 | 300642 | NM_014467.2 |
| BMPR1B | 603248 | NM_001203.2 | GATA4 | 600576 | NM_002052.4 | NOTCH2 | 600275 | NM_024408.3 | SSR4 | 300090 | NM_001204526.1 |
| BMPR2 | 600799 | NM_001204.6 | GATA5 | 611496 | NM_080473.4 | NOTCH3 | 600276 | NM_000435.2 | ST3GAL3 | 606494 | NM_006279.3 |
| BOLA3 | 613183 | NM_212552.2 | GATA6 | 601656 | NM_005257.5 | NPC1 | 607623 | NM_000271.4 | STAC3 | 615521 | NM_145064.2 |
| BRAF | 164757 | NM_004333.4 | GATAD2B | 614998 | NM_020699.2 | NPC2 | 601015 | NM_006432.3 | STAMBP | 606247 | NM_006463.4 |
| BRWD3 | 300553 | NM_153252.4 | GBA | 606463 | NM_001005741.2 | NPHP1 | 607100 | NM_000272.3 | STAT2 | 600556 | NM_005419.3 |
| BSCL2 | 606158 | NM_032667.6 | GBA2 | 609471 | NM_020944.2 | NPHP3 | 608002 | NM_153240.4 | STIL | 181590 | NM_003035.2 |
| BSND | 606412 | NM_057176.2 | GBE1 | 607839 | NM_000158.3 | NPHP4 | 607215 | NM_015102.4 | STIM1 | 605921 | NM_003156.3 |
| BTD | 609019 | NM_000060.3 | GCDH | 608801 | NM_000159.3 | NPHS1 | 602716 | NM_004646.3 | STRA6 | 610745 | NM_022369.3 |
| BVES | 604577 | NM_147147.3 | GCH1 | 600225 | NM_000161.2 | NPHS2 | 604766 | NM_014625.3 | STRADA | 608626 | NM_001003787.2 |
| C10ORF11 | 614537 | NM_032024.4 | GCK | 138079 | NM_000162.3 | NPRL3 | 600928 | NM_001243247.1 | STT3A | 601134 | NM_001278503.1 |
| C10ORF2-TWNK | 606075 | NM_021830.4 | GCNT2 | 600429 | NM_001491.2 | NR1H4 | 603826 | NM_005123.3 | STT3B | 608605 | NM_178862.2 |
| C12ORF57 | 615140 | NM_138425.3 | GCSH | 238330 | NM_004483.4 | NR2F2 | 107773 | NM_021005.3 | STUB1 | 607207 | NM_005861.3 |
| C12ORF65 | 613541 | NM_152269.4 | GDAP1 | 606598 | NM_018972.2 | NRAS | 164790 | NM_002524.4 | STX1B | 601485 | NM_052874.4 |
| C19ORF12 | 614297 | NM_001031726.3 | GDF1 | 602880 | NM_001492.5 | NRXN1 | 600565 | NM_001135659.1 | STXBP1 | 602926 | NM_003165.3 |
| C2CD3 | 615944 | NM_001286577.1 | GDF3 | 606522 | NM_020634.1 | NSD1 | 606681 | NM_022455.4 | SUCLA2 | 603921 | NM_003850.2 |
| C5ORF42 | 614571 | NM_023073.3 | GDF6 | 601147 | NM_001001557.2 | NSDHL | 300275 | NM_015922.2 | SUCLG1 | 611224 | NM_003849.3 |
| CACNA1A | 601011 | NM_001127221.1 | GDI1 | 300104 | NM_001493.2 | NSUN2 | 610916 | NM_017755.5 | SUMF1 | 607939 | NM_182760.3 |
| CACNA1B | 601012 | NM_000718.3 | GFAP | 137780 | NM_002055.4 | NT5C2 | 600417 | NM_012229.4 | SURF1 | 185620 | NM_003172.3 |
| CACNA1C | 114205 | NM_000719.6 | GFER | 600924 | NM_005262.2 | NTRK1 | 191315 | NM_001007792.1 | SYN1 | 313440 | NM_133499.2 |
| CACNA1G | 604065 | NM_018896.4 | GFM1 | 606639 | NM_024996.5 | NUBPL | 613621 | NM_025152.2 | SYNE1 | 608441 | NM_033071.3 |
| CACNA1H | 607904 | NM_021098.2 | GFPT1 | 138292 | NM_001244710.1 | NXF5 | 300319 | NM_032946.2 | SYNE2 | 608442 | NM_182914.2 |
| CACNA1S | 114208 | NM_000069.2 | GGPS1 | 606982 | NM_001037277.1 | OBSL1 | 610991 | NM_015311.2 | SYNGAP1 | 603384 | NM_006772.2 |
| CACNB4 | 601949 | NM_000726.3 | GJA1 | 121014 | NM_000165.4 | OCA2 | 611409 | NM_000275.2 | SYNJ1 | 604297 | NM_003895.3 |
| CACNG2 | 602911 | NM_006078.3 | GJA3 | 121015 | NM_021954.3 | OCLN | 602876 | NM_002538.3 | SYP | 313475 | NM_003179.2 |
| CAD | 114010 | NM_004341.4 | GJA8 | 600897 | NM_005267.4 | OCRL | 300535 | NM_000276.3 | SYT14 | 610949 | NM_001146261.2 |
| CALM1 | 114180 | NM_006888.4 | GJB1 | 304040 | NM_001097642.2 | OFD1 | 300170 | NM_003611.2 | SYT2 | 600104 | NM_177402.4 |
| CALM2 | 114182 | NM_001305624.1 | GJC2 | 608803 | NM_020435.3 | OPA1 | 605290 | NM_015560.2 | TAB2 | 605101 | NM_015093.5 |
| CAPN3 | 114240 | NM_000070.2 | GK | 300474 | NM_000167.5 | OPA3 | 606580 | NM_025136.3 | TACO1 | 612958 | NM_016360.3 |
| CARS2 | 612800 | NM_024537.3 | GLA | 300644 | NM_000169.2 | OPHN1 | 300127 | NM_002547.2 | TACSTD2 | 137290 | NM_002353.2 |
| CASK | 300172 | NM_003688.3 | GLB1 | 611458 | NM_000404.3 | OPTN | 602432 | NM_021980.4 | TAF1 | 313650 | NM_004606.4 |
| CASQ1 | 114250 | NM_001231.4 | GLDC | 238300 | NM_000170.2 | ORAI1 | 610277 | NM_032790.3 | TALDO1 | 602063 | NM_006755.1 |
| CASQ2 | 114251 | NM_001232.3 | GLE1 | 603371 | NM_001003722.1 | OTC | 300461 | NM_000531.5 | TANGO2 | 616830 | NM_152906.5 |
| CASR | 601199 | NM_000388.3 | GLI2 | 165230 | NM_005270.4 | OTX2 | 600037 | NM_172337.2 | TARDBP | 605078 | NM_007375.3 |
| CAV1 | 601047 | NM_001753.4 | GLI3 | 165240 | NM_000168.5 | OVOL2 | 616441 | NM_021220.3 | TARS2 | 612805 | NM_025150.4 |
| CAV3 | 601253 | NM_033337.2 | GLRA1 | 138491 | NM_000171.3 | P3H2 | 610341 | NM_018192.3 | TAZ | 300394 | NM_000116.4 |
| CBL | 165360 | NM_005188.3 | GLRB | 138492 | NM_000824.4 | PABPN1 | 602279 | NM_004643.3 | TBC1D20 | 611663 | NM_144628.3 |
| CBS | 613381 | NM_000071.2 | GMPPA | 615495 | NM_205847.2 | PACS1 | 607492 | NM_018026.3 | TBC1D24 | 613577 | NM_001199107.1 |
| CC2D1A | 610055 | NM_017721.4 | GMPPB | 615320 | NM_021971.2 | PAFAH1B1 | 601545 | NM_000430.3 | TBC1D7 | 612655 | NM_001143965.3 |
| CC2D2A | 612013 | NM_001080522.2 | GNAL | 139312 | NM_001142339.2 | PAK3 | 300142 | NM_002578.4 | TBK1 | 604834 | NM_013254.3 |
| CCDC115 | 613734 | NM_032357.3 | GNAQ | 600998 | NM_002072.4 | PANK2 | 606157 | NM_153638.3 | TBX1 | 602054 | NM_080647.1 |
| CCDC22 | 300859 | NM_014008.4 | GNAS | 139320 | NM_000516.5 | PARK2 | 602544 | NM_004562.2 | TBX20 | 606061 | NM_001077653.2 |
| CCDC78 | 614666 | NM_001031737.2 | GNB4 | 610863 | NM_021629.3 | PARK7 | 602533 | NM_007262.4 | TBX3 | 601621 | NM_005996.3 |
| CCDC8 | 614145 | NM_032040.4 | GNE | 603824 | NM_005476.5 | PARS2 | 612036 | NM_152268.3 | TBX4 | 601719 | NM_018488.3 |
| CCND2 | 123833 | NM_001759.3 | GNPTAB | 607840 | NM_024312.4 | PAX2 | 167409 | NM_003987.4 | TBX5 | 601620 | NM_000192.3 |
| CCNO | 607752 | NM_021147.4 | GNPTG | 607838 | NM_032520.4 | PAX3 | 606597 | NM_181457.3 | TCAP | 604488 | NM_003673.3 |
| CCT5 | 610150 | NM_012073.4 | GNS | 607664 | NM_002076.3 | PAX4 | 167413 | NM_006193.2 | TCF4 | 602272 | NM_001083962.1 |
| CD2AP | 604241 | NM_012120.2 | GOSR2 | 604027 | NM_004287.4 | PAX6 | 607108 | NM_000280.4 | TCOF1 | 606847 | NM_001135243.1 |
| CDC5L | 602868 | NM_001253.3 | GPC3 | 300037 | NM_004484.3 | PAX8 | 167415 | NM_003466.3 | TCTN3 | 613847 | NM_015631.5 |
| CDH15 | 114019 | NM_004933.2 | GPHN | 603930 | NM_020806.4 | PC | 608786 | NM_000920.3 | TDGF1 | 187395 | NM_003212.3 |
| CDK5 | 123831 | NM_004935.3 | GPR143 | 300808 | NM_000273.2 | PCCA | 232000 | NM_000282.3 | TDP1 | 607198 | NM_018319.3 |
| CDK5RAP2 | 608201 | NM_018249.5 | GPSM2 | 609245 | NM_013296.4 | PCCB | 232050 | NM_000532.4 | TDRD7 | 611258 | NM_014290.2 |
| CDKL5 | 300203 | NM_003159.2 | GREM1 | 603054 | NM_013372.6 | PCDH19 | 300460 | NM_001184880.1 | TECPR2 | 615000 | NM_014844.4 |
| CDKN1C | 600856 | NM_000076.2 | GRHPR | 604296 | NM_012203.1 | PDE8B | 603390 | NM_003719.3 | TECR | 610057 | NM_138501.5 |
| CDON | 608707 | NM_016952.4 | GRIA3 | 305915 | NM_000828.4 | PDGFB | 190040 | NM_002608.3 | TENM3 | 610083 | NM_001080477.3 |
| CECR1 | 607575 | NM_001282225.1 | GRID2 | 602368 | NM_001510.3 | PDHA1 | 300502 | NM_000284.3 | TFAP2A | 107580 | NM_003220.2 |
| CEL | 114840 | NM_001807.4 | GRIK2 | 138244 | NM_001166247.1 | PDHB | 179060 | NM_000925.3 | TFG | 602498 | NM_006070.5 |
| CENPJ | 609279 | NM_018451.4 | GRIN1 | 138249 | NM_007327.3 | PDHX | 608769 | NM_003477.2 | TGFB2 | 190220 | NM_003238.3 |
| CEP152 | 613529 | NM_014985.3 | GRIN2A | 138253 | NM_000833.4 | PDK3 | 300906 | NM_001142386.2 | TGFB3 | 190230 | NM_003239.3 |
| CFC1 | 605194 | NM_001270420.1 | GRIN2B | 138252 | NM_000834.3 | PDP1 | 605993 | NM_018444.3 | TGFBI | 601692 | NM_000358.2 |
| CFL2 | 601443 | NM_138638.4 | GRIP1 | 604597 | NM_021150.3 | PDSS1 | 607429 | NM_014317.4 | TGFBR1 | 190181 | NM_004612.3 |
| CFTR | 602421 | NM_000492.3 | GRM1 | 604473 | NM_001278065.1 | PDSS2 | 610564 | NM_020381.3 | TGFBR2 | 190182 | NM_001024847.2 |
| CHAT | 118490 | NM_020549.4 | GRN | 138945 | NM_002087.3 | PDX1 | 600733 | NM_000209.3 | TGIF1 | 602630 | NM_173208.2 |
| CHCHD10 | 615903 | NM_213720.2 | GSN | 137350 | NM_000177.4 | PDYN | 131340 | NM_024411.4 | TGM6 | 613900 | NM_198994.2 |
| CHD1L | 613039 | NM_004284.4 | GTPBP2 | 607434 | NM_001286216.1 | PEX1 | 602136 | NM_000466.2 | TH | 191290 | NM_199292.2 |
| CHD2 | 602119 | NM_001271.3 | GTPBP3 | 608536 | NM_001195422.1 | PEX10 | 602859 | NM_153818.1 | THAP1 | 609520 | NM_018105.2 |
| CHD7 | 608892 | NM_017780.3 | GUSB | 611499 | NM_000181.3 | PEX11B | 603867 | NM_003846.2 | THOC2 | 300395 | NM_001081550.1 |
| CHD8 | 610528 | NM_001170629.1 | GYG1 | 603942 | NM_004130.3 | PEX12 | 601758 | NM_000286.2 | TIA1 | 603518 | NM_022173.2 |
| CHKB | 612395 | NM_005198.4 | HADH | 601609 | NM_005327.4 | PEX13 | 601789 | NM_002618.3 | TIMM8A | 300356 | NM_004085.3 |
| CHMP2B | 609512 | NM_014043.3 | HADHA | 600890 | NM_000182.4 | PEX14 | 601791 | NM_004565.2 | TJP2 | 607709 | NM_004817.3 |
| CHMP4B | 610897 | NM_176812.4 | HADHB | 143450 | NM_000183.2 | PEX16 | 603360 | NM_004813.2 | TK2 | 188250 | NM_004614.4 |
| CHRM3 | 118494 | NM_000740.2 | HARS | 142810 | NM_002109.5 | PEX19 | 600279 | NM_002857.3 | TLL1 | 606742 | NM_012464.4 |
| CHRNA1 | 100690 | NM_000079.3 | HARS2 | 600783 | NM_012208.3 | PEX2 | 170993 | NM_000318.2 | TMEM114 | 611579 | NM_001290097.1 |
| CHRNA2 | 118502 | NM_000742.3 | HCCS | 300056 | NM_005333.4 | PEX26 | 608666 | NM_017929.5 | TMEM126A | 612988 | NM_032273.3 |
| CHRNA4 | 118504 | NM_000744.6 | HCFC1 | 300019 | NM_005334.2 | PEX3 | 603164 | NM_003630.2 | TMEM165 | 614726 | NM_018475.4 |
| CHRNA7 | 118511 | NM_000746.5 | HCN1 | 602780 | NM_021072.3 | PEX5 | 600414 | NM_001131025.1 | TMEM216 | 613277 | NM_001173990.2 |
| CHRNB1 | 100710 | NM_000747.2 | HDAC8 | 300269 | NM_018486.2 | PEX6 | 601498 | NM_000287.3 | TMEM43 | 612048 | NM_024334.2 |
| CHRNB2 | 118507 | NM_000748.2 | HEPACAM | 611642 | NM_152722.4 | PEX7 | 601757 | NM_000288.3 | TMEM5 | 605862 | NM_014254.2 |
| CHRND | 100720 | NM_000751.2 | HERC2 | 605837 | NM_004667.5 | PFKM | 610681 | NM_000289.5 | TMEM70 | 612418 | NM_017866.5 |
| CHRNE | 100725 | NM_000080.3 | HESX1 | 601802 | NM_003865.2 | PGAP1 | 611655 | NM_024989.3 | TMEM98 | 615949 | NM_001301746.1 |
| CHRNG | 100730 | NM_005199.4 | HEXA | 606869 | NM_000520.5 | PGK1 | 311800 | NM_000291.3 | TNNC1 | 191040 | NM_003280.2 |
| CHST14 | 608429 | NM_130468.3 | HEXB | 606873 | NM_000521.3 | PGM1 | 171900 | NM_002633.2 | TNNI2 | 191043 | NM_003282.3 |
| CHST6 | 605294 | NM_021615.4 | HFE | 613609 | NM_000410.3 | PHF6 | 300414 | NM_001015877.1 | TNNI3 | 191044 | NM_000363.4 |
| CISD2 | 611507 | NM_001008388.4 | HGSNAT | 610453 | NM_152419.2 | PHF8 | 300560 | NM_015107.2 | TNNT1 | 191041 | NM_003283.5 |
| CITED2 | 602937 | NM_006079.4 | HIBCH | 610690 | NM_014362.3 | PHGDH | 606879 | NM_006623.3 | TNNT2 | 191045 | NM_001001430.2 |
| CIZ1 | 611420 | NM_001131017.1 | HINT1 | 601314 | NM_005340.6 | PHKA1 | 311870 | NM_002637.3 | TNNT3 | 600692 | NM_006757.3 |
| CLCN1 | 118425 | NM_000083.2 | HK1 | 142600 | NM_033500.2 | PHKA2 | 300798 | NM_000292.2 | TNPO3 | 610032 | NM_012470.3 |
| CLCN2 | 600570 | NM_004366.5 | HMBS | 609806 | NM_000190.3 | PHKB | 172490 | NM_000293.2 | TNXB | 600985 | NM_019105.6 |
| CLCN4 | 302910 | NM_001830.3 | HMX1 | 142992 | NM_018942.2 | PHKG2 | 172471 | NM_000294.2 | TOR1A | 605204 | NM_000113.2 |
| CLCN5 | 300008 | NM_000084.4 | HNF1A | 142410 | NM_000545.6 | PHOX2A | 602753 | NM_005169.3 | TOR1AIP1 | 614512 | NM_001267578.1 |
| CLCNKA | 602024 | NM_004070.3 | HNF1B | 189907 | NM_000458.3 | PHOX2B | 603851 | NM_003924.3 | TP63 | 603273 | NM_003722.4 |
| CLCNKB | 602023 | NM_000085.4 | HNF4A | 600281 | NM_175914.4 | PHYH | 602026 | NM_006214.3 | TPK1 | 606370 | NM_022445.3 |
| CLDN1 | 603718 | NM_021101.4 | HNRNPA1 | 164017 | NM_031157.3 | PIEZO2 | 613629 | NM_022068.3 | TPM1 | 191010 | NM_001018005.1 |
| CLIC2 | 300138 | NM_001289.5 | HNRNPA2B1 | 600124 | NM_031243.2 | PIK3CA | 171834 | NM_006218.3 | TPM2 | 190990 | NM_213674.1 |
| CLN3 | 607042 | NM_000086.2 | HNRNPDL | 607137 | NM_031372.3 | PIK3R2 | 603157 | NM_005027.3 | TPM3 | 191030 | NM_152263.3 |
| CLN5 | 608102 | NM_006493.2 | HNRNPU | 602869 | NM_031844.2 | PIK3R5 | 611317 | NM_001142633.2 | TPP1 | 607998 | NM_000391.3 |
| CLN6 | 606725 | NM_017882.2 | HOGA1 | 613597 | NM_138413.3 | PIKFYVE | 609414 | NM_015040.3 | TRAP1 | 606219 | NM_016292.2 |
| CLN8 | 607837 | NM_018941.3 | HOXA1 | 142955 | NM_005522.4 | PINK1 | 608309 | NM_032409.2 | TRAPPC11 | 614138 | NM_021942.5 |
| CLP1 | 608757 | NM_006831.2 | HOXD13 | 142989 | NM_000523.3 | PIP5K1C | 606102 | NM_012398.2 | TRAPPC9 | 611966 | NM_031466.7 |
| CLPB | 616254 | NM_001258394.2 | HPCA | 142622 | NM_002143.2 | PITRM1 | - | NM_001242307.1 | TRDN | 603283 | NM_006073.3 |
| CLPP | 601119 | NM_006012.2 | HPRT1 | 308000 | NM_000194.2 | PITX1 | 602149 | NM_002653.4 | TREX1 | 606609 | NM_033629.4 |
| CNTN1 | 600016 | NM_001843.3 | HPSE2 | 613469 | NM_021828.4 | PITX2 | 601542 | NM_153427.2 | TRIM2 | 614141 | NM_015271.4 |
| CNTNAP1 | 602346 | NM_003632.2 | HRAS | 190020 | NM_005343.3 | PKD2 | 173910 | NM_000297.3 | TRIM32 | 602290 | NM_012210.3 |
| CNTNAP2 | 604569 | NM_014141.5 | HSD17B10 | 300256 | NM_004493.2 | PKHD1 | 606702 | NM_138694.3 | TRIM37 | 605073 | NM_015294.4 |
| COA5 | 613920 | NM_001008215.2 | HSD17B4 | 601860 | NM_000414.3 | PKP2 | 602861 | NM_004572.3 | TRIP4 | 604501 | NM_016213.4 |
| COASY | 609855 | NM_001042532.3 | HSD3B7 | 607764 | NM_025193.3 | PLA2G6 | 603604 | NM_003560.2 | TRIT1 | 617840 | NM_017646.5 |
| COG1 | 606973 | NM_018714.2 | HSF4 | 602438 | NM_001538.3 | PLCB1 | 607120 | NM_015192.3 | TRMT5 | 611023 | NM_020810.3 |
| COG4 | 606976 | NM_015386.2 | HSPB1 | 602195 | NM_001540.3 | PLCE1 | 608414 | NM_016341.3 | TRMU | 610230 | NM_018006.4 |
| COG5 | 606821 | NM_006348.3 | HSPB3 | 604624 | NM_006308.2 | PLEC | 601282 | NM_000445.4 | TRNT1 | 612907 | NM_001302946.1 |
| COG6 | 606977 | NM_020751.2 | HSPB8 | 608014 | NM_014365.2 | PLEKHG5 | 611101 | NM_020631.4 | TRPA1 | 604775 | NM_007332.2 |
| COG7 | 606978 | NM_153603.3 | HSPD1 | 118190 | NM_002156.4 | PLN | 172405 | NM_002667.4 | TRPC6 | 603652 | NM_004621.5 |
| COG8 | 606979 | NM_032382.4 | HSPG2 | 142461 | NM_005529.6 | PLOD1 | 153454 | NM_000302.3 | TRPS1 | 604386 | NM_014112.4 |
| COL11A1 | 120280 | NM_001854.3 | HTRA1 | 602194 | NM_002775.4 | PLOD2 | 601865 | NM_182943.2 | TRPV4 | 605427 | NM_021625.4 |
| COL11A2 | 120290 | NM_080680.2 | HUWE1 | 300697 | NM_031407.6 | PLP1 | 300401 | NM_000533.4 | TSC1 | 605284 | NM_000368.4 |
| COL12A1 | 120320 | NM_004370.5 | IARS2 | 612801 | NM_018060.3 | PLXND1 | 604282 | NM_015103.2 | TSC2 | 191092 | NM_000548.4 |
| COL13A1 | 120350 | NM_001130103.1 | IBA57 | 615316 | NM_001010867.3 | PMM2 | 601785 | NM_000303.2 | TSEN2 | 608753 | NM_025265.3 |
| COL17A1 | 113811 | NM_000494.3 | IDH1 | 147700 | NM_005896.3 | PMP2 | 170715 | NM_002677.3 | TSEN34 | 608754 | NM_024075.4 |
| COL18A1 | 120328 | NM_130445.3 | IDH2 | 147650 | NM_001289910.1 | PMP22 | 601097 | NM_000304.3 | TSEN54 | 608755 | NM_207346.2 |
| COL1A1 | 120150 | NM_000088.3 | IDS | 300823 | NM_000202.7 | PMPCA | 613036 | NM_015160.2 | TSFM | 604723 | NM_001172696.1 |
| COL1A2 | 120160 | NM_000089.3 | IDUA | 252800 | NM_000203.4 | PNKD | 609023 | NM_015488.4 | TSPAN7 | 300096 | NM_004615.3 |
| COL2A1 | 120140 | NM_001844.4 | IFIH1 | 606951 | NM_022168.3 | PNKP | 605610 | NM_007254.3 | TTBK2 | 611695 | NM_173500.3 |
| COL3A1 | 120180 | NM_000090.3 | IFRD1 | 603502 | NM_001550.3 | PNPLA2 | 609059 | NM_020376.3 | TTC19 | 613814 | NM_017775.3 |
| COL4A1 | 120130 | NM_001845.5 | IGBP1 | 300139 | NM_001551.2 | PNPLA6 | 603197 | NM_006702.4 | TTN | 188840 | NM_001267550.2 |
| COL4A2 | 120090 | NM_001846.2 | IGHMBP2 | 600502 | NM_002180.2 | PNPO | 603287 | NM_018129.3 | TTPA | 600415 | NM_000370.3 |
| COL4A3 | 120070 | NM_000091.4 | IKBKAP | 603722 | NM_003640.4 | PNPT1 | 610316 | NM_033109.4 | TTR | 176300 | NM_000371.3 |
| COL4A4 | 120131 | NM_000092.4 | IKBKG | 300248 | NM_003639.4 | PODXL | 602632 | NM_001018111.2 | TUBA1A | 602529 | NM_006009.3 |
| COL4A5 | 303630 | NM_000495.4 | IL1RAPL1 | 300206 | NM_014271.3 | POLG | 174763 | NM_002693.2 | TUBA4A | 191110 | NM_006000.2 |
| COL5A1 | 120215 | NM_000093.4 | INF2 | 610982 | NM_022489.3 | POLG2 | 604983 | NM_007215.3 | TUBA8 | 605742 | NM_018943.2 |
| COL5A2 | 120190 | NM_000393.3 | INS | 176730 | NM_001185098.1 | POLR1C | 610060 | NM_203290.3 | TUBB | 191130 | NM_178014.3 |
| COL6A1 | 120220 | NM_001848.2 | INSR | 147670 | NM_000208.3 | POLR1D | 613715 | NM_015972.3 | TUBB2A | 615101 | NM_001069.2 |
| COL6A2 | 120240 | NM_001849.3 | INVS | 243305 | NM_014425.4 | POLR3A | 614258 | NM_007055.3 | TUBB2B | 612850 | NM_178012.4 |
| COL6A3 | 120250 | NM_004369.3 | IQSEC2 | 300522 | NM_001111125.2 | POLR3B | 614366 | NM_018082.5 | TUBB3 | 602661 | NM_006086.3 |
| COL8A2 | 120252 | NM_005202.3 | IRF6 | 607199 | NM_006147.3 | POMGNT1 | 606822 | NM_017739.3 | TUBB4A | 602662 | NM_006087.3 |
| COL9A1 | 120210 | NM_001851.4 | ISCA2 | 615317 | NM_194279.3 | POMGNT2 | 614828 | NM_032806.5 | TUBG1 | 191135 | NM_001070.4 |
| COL9A2 | 120260 | NM_001852.3 | ISCU | 611911 | NM_213595.3 | POMK | 615247 | NM_032237.4 | TUBG2 | 605785 | NM_001320509.1 |
| COL9A3 | 120270 | NM_001853.3 | ISPD | 614631 | NM_001101426.3 | POMT1 | 607423 | NM_007171.3 | TUFM | 602389 | NM_003321.4 |
| COLQ | 603033 | NM_005677.3 | ITGA3 | 605025 | NM_002204.3 | POMT2 | 607439 | NM_013382.5 | TUSC3 | 601385 | NM_006765.3 |
| COQ2 | 609825 | NM_015697.7 | ITGA7 | 600536 | NM_002206.2 | POR | 124015 | NM_000941.2 | TWIST1 | 601622 | NM_000474.3 |
| COQ4 | 612898 | NM_016035.4 | ITGA8 | 604063 | NM_003638.2 | PORCN | 300651 | NM_203475.2 | TXN2 | 609063 | NM_012473.3 |
| COQ6 | 614647 | NM_182476.2 | ITM2B | 603904 | NM_021999.4 | PPOX | 600923 | NM_000309.3 | TXNL4A | 611595 | NM_006701.3 |
| COQ7 | 601683 | NM_016138.4 | ITPR1 | 147265 | NM_002222.5 | PPP2R2B | 604325 | NM_181675.3 | TYMP | 131222 | NM_001257988.1 |
| COQ9 | 612837 | NM_020312.3 | IVD | 607036 | NM_002225.3 | PPT1 | 600722 | NM_000310.3 | TYR | 606933 | NM_000372.4 |
| COX10 | 602125 | NM_001303.3 | JAG1 | 601920 | NM_000214.2 | PPT2 | 603298 | NM_138717.2 | TYRP1 | 115501 | NM_000550.2 |
| COX14 | 614478 | NM_032901.3 | JAM3 | 606871 | NM_032801.4 | PQBP1 | 300463 | NM_005710.2 | UBA1 | 314370 | NM_003334.3 |
| COX15 | 603646 | NM_078470.5 | JUP | 173325 | NM_021991.2 | PRDM5 | 614161 | NM_018699.3 | UBA5 | 610552 | NM_024818.4 |
| COX4I2 | 607976 | NM_032609.2 | KANSL1 | 612452 | NM_001193466.1 | PRDM8 | 616639 | NM_020226.3 | UBE2A | 312180 | NM_003336.3 |
| COX6A1 | 602072 | NM_004373.3 | KARS | 601421 | NM_001130089.1 | PREPL | 609557 | NM_006036.4 | UBE3A | 601623 | NM_130838.1 |
| COX6B1 | 124089 | NM_001863.4 | KAT6A | 601408 | NM_006766.4 | PRICKLE1 | 608500 | NM_153026.2 | UBIAD1 | 611632 | NM_013319.2 |
| COX8A | 123870 | NM_004074.2 | KAT6B | 605880 | NM_012330.3 | PRICKLE2 | 608501 | NM_198859.3 | UBQLN2 | 300264 | NM_013444.3 |
| CP | 117700 | NM_000096.3 | KATNB1 | 602703 | NM_005886.2 | PRIMA1 | 613851 | NM_178013.3 | UBR1 | 605981 | NM_174916.2 |
| CPOX | 612732 | NM_000097.5 | KBTBD13 | 613727 | NM_001101362.2 | PRKAG2 | 602743 | NM_016203.3 | UGT1A1 | 191740 | NM_000463.2 |
| CPS1 | 608307 | NM_001875.4 | KCNA1 | 176260 | NM_000217.2 | PRKCG | 176980 | NM_002739.4 | UMOD | 191845 | NM_003361.3 |
| CPT1A | 600528 | NM_001876.3 | KCNA2 | 176262 | NM_001204269.1 | PRKCSH | 177060 | NM_002743.3 | UPF3B | 300298 | NM_080632.2 |
| CPT1C | 608846 | NM_001136052.2 | KCNA4 | 176266 | NM_002233.3 | PRKG1 | 176894 | NM_006258.3 | UPK2 | 611558 | NM_006760.3 |
| CPT2 | 600650 | NM_000098.2 | KCNB1 | 600397 | NM_004975.3 | PRKRA | 603424 | NM_003690.4 | UPK3A | 611559 | NM_006953.3 |
| CRB2 | 609720 | NM_173689.6 | KCNC1 | 176258 | NM_001112741.1 | PRNP | 176640 | NM_000311.3 | UQCRB | 191330 | NM_006294.4 |
| CRBN | 609262 | NM_016302.3 | KCNC3 | 176264 | NM_004977.2 | PRPS1 | 311850 | NM_002764.3 | UQCRC2 | 191329 | NM_003366.3 |
| CREBBP | 600140 | NM_004380.2 | KCND3 | 605411 | NM_004980.4 | PRRT2 | 614386 | NM_145239.2 | UQCRQ | 612080 | NM_014402.4 |
| CRELD1 | 607170 | NM_015513.4 | KCNE1 | 176261 | NM_000219.5 | PRSS56 | 613858 | NM_001195129.1 | UROD | 613521 | NM_000374.4 |
| CRYAA | 123580 | NM_000394.3 | KCNE2 | 603796 | NM_172201.1 | PRX | 605725 | NM_020956.2 | UROS | 606938 | NM_000375.2 |
| CRYAB | 123590 | NM_001885.2 | KCNE3 | 604433 | NM_005472.4 | PSAP | 176801 | NM_002778.3 | USP8 | 603158 | NM_001128610.2 |
| CRYBA1 | 123610 | NM_005208.4 | KCNH2 | 152427 | NM_000238.3 | PSAT1 | 610936 | NM_058179.3 | UTP4 | 607456 | NM_032830.2 |
| CRYBA4 | 123631 | NM_001886.2 | KCNH5 | 605716 | NM_139318.4 | PSEN1 | 104311 | NM_000021.3 | UTRN | 128240 | NM_007124.2 |
| CRYBB1 | 600929 | NM_001887.3 | KCNJ1 | 600359 | NM_000220.4 | PSEN2 | 600759 | NM_000447.2 | VAMP1 | 185880 | NM_014231.4 |
| CRYBB2 | 123620 | NM_000496.2 | KCNJ10 | 602208 | NM_002241.4 | PTCD1 | 614774 | NM_015545.3 | VAPB | 605704 | NM_004738.4 |
| CRYBB3 | 123630 | NM_004076.4 | KCNJ11 | 600937 | NM_000525.3 | PTCH1 | 601309 | NM_000264.3 | VARS2 | 612802 | NM_001167734.1 |
| CRYGB | 123670 | NM_005210.3 | KCNJ2 | 600681 | NM_000891.2 | PTCHD1 | 300828 | NM_173495.2 | VAX1 | 604294 | NM_001112704.1 |
| CRYGC | 123680 | NM_020989.3 | KCNJ5 | 600734 | NM_000890.3 | PTEN | 601728 | NM_000314.6 | VCAN | 118661 | NM_004385.4 |
| CRYGD | 123690 | NM_006891.3 | KCNK3 | 603220 | NM_002246.2 | PTPN11 | 176876 | NM_002834.3 | VCL | 193065 | NM_014000.2 |
| CRYGS | 123730 | NM_017541.2 | KCNMA1 | 600150 | NM_002247.3 | PTPRO | 600579 | NM_030667.2 | VCP | 601023 | NM_007126.3 |
| CSF1R | 164770 | NM_005211.3 | KCNQ1 | 607542 | NM_000218.2 | PTRF | 603198 | NM_012232.5 | VHL | 608537 | NM_000551.3 |
| CSRP3 | 600824 | NM_003476.4 | KCNQ2 | 602235 | NM_172107.3 | PTRH2 | 608625 | NM_001015509.2 | VIM | 193060 | NM_003380.3 |
| CST3 | 604312 | NM_000099.3 | KCNQ3 | 602232 | NM_004519.3 | PUS1 | 608109 | NM_025215.5 | VIPAS39 | 613401 | NM_001193315.1 |
| CSTB | 601145 | NM_000100.3 | KCNT1 | 608167 | NM_020822.2 | PXDN | 605158 | NM_012293.2 | VLDLR | 192977 | NM_003383.4 |
| CTC1 | 613129 | NM_025099.5 | KCTD17 | 616386 | NM_001282684.1 | PYCR2 | 616406 | NM_013328.3 | VMA21 | 300913 | NM_001017980.3 |
| CTCF | 604167 | NM_006565.3 | KCTD7 | 611725 | NM_153033.4 | PYGL | 613741 | NM_002863.4 | VPS11 | 608549 | NM_001290185.1 |
| CTDP1 | 604927 | NM_004715.4 | KDM5C | 314690 | NM_004187.3 | PYGM | 608455 | NM_005609.3 | VPS13A | 605978 | NM_033305.2 |
| CTNNB1 | 116806 | NM_001904.3 | KDM6A | 300128 | NM_001291415.1 | QARS | 603727 | NM_005051.2 | VPS13B | 607817 | NM_017890.4 |
| CTSC | 602365 | NM_001814.5 | KIAA0196 | 610657 | NM_014846.3 | RAB18 | 602207 | NM_021252.4 | VPS33B | 608552 | NM_018668.4 |
| CTSF | 603539 | NM_003793.3 | KIAA2022 | 300524 | NM_001008537.2 | RAB23 | 606144 | NM_183227.2 | VPS35 | 601501 | NM_018206.5 |
| CUL3 | 603136 | NM_003590.4 | KIF1A | 601255 | NM_004321.6 | RAB39B | 300774 | NM_171998.3 | VPS37A | 609927 | NM_152415.2 |
| CUL4B | 300304 | NM_003588.3 | KIF1BP | 609367 | NM_015634.3 | RAB3GAP1 | 602536 | NM_012233.2 | VRK1 | 602168 | NM_003384.2 |
| CUL7 | 609577 | NM_014780.4 | KIF1C | 603060 | NM_006612.5 | RAB3GAP2 | 609275 | NM_012414.3 | VSX1 | 605020 | NM_014588.5 |
| CWF19L1 | 616120 | NM_001303405.1 | KIF21A | 608283 | NM_017641.3 | RAB40AL | 300405 | NM_001031834.1 | VSX2 | 142993 | NM_182894.2 |
| CYP1B1 | 601771 | NM_000104.3 | KIF2A | 602591 | NM_001098511.2 | RAB7A | 602298 | NM_004637.5 | WAC | 615049 | NM_016628.4 |
| CYP24A1 | 126065 | NM_000782.4 | KIF5A | 602821 | NM_004984.2 | RAD21 | 606462 | NM_006265.2 | WDR45 | 300526 | NM_007075.3 |
| CYP27A1 | 606530 | NM_000784.3 | KIF5C | 604593 | NM_004522.2 | RAF1 | 164760 | NM_002880.3 | WDR48 | 612167 | NM_001303403.1 |
| CYP2U1 | 610670 | NM_183075.2 | KIF7 | 611254 | NM_198525.2 | RAI1 | 607642 | NM_030665.3 | WDR62 | 613583 | NM_001083961.1 |
| CYP4V2 | 608614 | NM_207352.3 | KLC4 | - | NM_201523.2 | RANBP2 | 601181 | NM_006267.4 | WFS1 | 606201 | NM_006005.3 |
| CYP7B1 | 603711 | NM_004820.4 | KLF11 | 603301 | NM_003597.4 | RAPSN | 601592 | NM_005055.4 | WNK1 | 605232 | NM_213655.4 |
| D2HGDH | 609186 | NM_152783.4 | KLF8 | 300286 | NM_007250.5 | RARB | 180220 | NM_001290217.1 | WNK4 | 601844 | NM_032387.4 |
| DACH1 | 603803 | NM_080759.5 | KLHL13 | 300655 | NM_033495.3 | RARS | 107820 | NM_002887.3 | WNT4 | 603490 | NM_030761.4 |
| DAG1 | 128239 | NM_004393.5 | KLHL3 | 605775 | NM_017415.2 | RARS2 | 611524 | NM_020320.4 | WNT5A | 164975 | NM_003392.4 |
| DARS | 603084 | NM_001349.3 | KLHL40 | 615340 | NM_152393.3 | RASA1 | 139150 | NM_002890.2 | WT1 | 607102 | NM_024426.4 |
| DARS2 | 610956 | NM_018122.4 | KLHL41 | 607701 | NM_006063.2 | RASA2 | 601589 | NM_006506.3 | WWOX | 605131 | NM_016373.3 |
| DBT | 248610 | NM_001918.3 | KMT2A | 159555 | NM_001197104.1 | RAX | 601881 | NM_013435.2 | XK | 314850 | NM_021083.2 |
| DCAF17 | 612515 | NM_025000.3 | KMT2D | 602113 | NM_003482.3 | RBCK1 | 610924 | NM_031229.3 | XPNPEP3 | 613553 | NM_022098.3 |
| DCAF8 | 615820 | NM_015726.3 | KPTN | 615620 | NM_007059.3 | RBM10 | 300080 | NM_005676.4 | XRCC4 | 194363 | NM_022406.3 |
| DCHS1 | 603057 | NM_003737.3 | KRAS | 190070 | NM_004985.4 | RBM20 | 613171 | NM_001134363.2 | YARS | 603623 | NM_003680.3 |
| DCN | 125255 | NM_001920.4 | KRT3 | 148043 | NM_057088.2 | RBPJ | 147183 | NM_005349.3 | YARS2 | 610957 | NM_001040436.2 |
| DCTN1 | 601143 | NM_004082.4 | KY | 605739 | NM_178554.4 | RECQL4 | 603780 | NM_004260.3 | ZBTB42 | 613915 | NM_001137601.2 |
| DCX | 300121 | NM_178153.2 | L1CAM | 308840 | NM_000425.4 | REEP1 | 609139 | NM_001164730.1 | ZC4H2 | 300897 | NM_018684.3 |
| DDHD1 | 614603 | NM_001160148.1 | L2HGDH | 609584 | NM_024884.2 | REEP2 | 609347 | NM_001271803.1 | ZDHHC15 | 300576 | NM_144969.2 |
| DDHD2 | 615003 | NM_015214.2 | LAMA2 | 156225 | NM_000426.3 | RELN | 600514 | NM_005045.3 | ZDHHC9 | 300646 | NM_016032.3 |
| DDOST | 602202 | NM_005216.4 | LAMB1 | 150240 | NM_002291.2 | REN | 179820 | NM_000537.3 | ZEB1 | 189909 | NM_030751.5 |
| DDX3X | 300160 | NM_001193416.2 | LAMB2 | 150325 | NM_002292.3 | RET | 164761 | NM_020975.4 | ZEB2 | 605802 | NM_014795.3 |
| DEPDC5 | 614191 | NM_001242896.1 | LAMP2 | 309060 | NM_002294.2 | REV3L | 602776 | NM_002912.4 | ZFPM2 | 603693 | NM_012082.3 |
| DES | 125660 | NM_001927.3 | LARGE1 | 603590 | NM_004737.4 | RFT1 | 611908 | NM_052859.3 | ZFR | 615635 | NM_016107.3 |
| DGAT2 | 606983 | NM_032564.4 | LARS | 151350 | NM_020117.10 | RIPK4 | 605706 | NM_020639.2 | ZFYVE26 | 612012 | NM_015346.3 |
| DGKE | 601440 | NM_003647.2 | LARS2 | 604544 | NM_015340.3 | RIT1 | 609591 | NM_001256821.1 | ZFYVE27 | 610243 | NM_001002261.3 |
| DGUOK | 601465 | NM_080916.2 | LAS1L | 300964 | NM_031206.4 | RMND1 | 614917 | NM_017909.3 | ZIC2 | 603073 | NM_007129.3 |
| DHCR7 | 602858 | NM_001360.2 | LDB3 | 605906 | NM_007078.2 | RNASEH1 | 604123 | NM_001286834.1 | ZIC3 | 300265 | NM_003413.3 |
| DHDDS | 608172 | NM_024887.3 | LGI1 | 604619 | NM_005097.3 | RNASEH2A | 606034 | NM_006397.2 | ZMPSTE24 | 606480 | NM_005857.4 |
| DHODH | 126064 | NM_001361.4 | LIAS | 607031 | NM_006859.3 | RNASEH2B | 610326 | NM_001142279.2 | ZMYM3 | 300061 | NM_201599.2 |
| DHTKD1 | 614984 | NM_018706.6 | LIM2 | 154045 | NM_030657.3 | RNASEH2C | 610330 | NM_032193.3 | ZNF41 | 314995 | NM_153380.3 |
| DIS3L2 | 614184 | NM_152383.4 | LIMS2 | 607908 | NM_001136037.2 | RNASET2 | 612944 | NM_003730.4 | ZNF469 | 612078 | NM_001127464.2 |
| DISP1 | 607502 | NM_032890.3 | LIPA | 613497 | NM_000235.3 | RNF135 | 611358 | NM_032322.3 | ZNF674 | 300573 | NM_001039891.2 |
| DKC1 | 300126 | NM_001363.4 | LITAF | 603795 | NM_001136473.1 | RNF216 | 609948 | NM_207111.3 | ZNF711 | 314990 | NM_021998.4 |
| DLAT | 608770 | NM_001931.4 | LMBR1 | 605522 | NM_022458.3 | ROBO2 | 602431 | NM_002942.4 | ZNF81 | 314998 | NM_007137.3 |
